# Mitochondrial fusion is required for regulation of mitochondrial DNA replication

**DOI:** 10.1371/journal.pgen.1008085

**Published:** 2019-06-06

**Authors:** Eduardo Silva Ramos, Elisa Motori, Christian Brüser, Inge Kühl, Assa Yeroslaviz, Benedetta Ruzzenente, Johanna H. K. Kauppila, Jakob D. Busch, Kjell Hultenby, Bianca H. Habermann, Stefan Jakobs, Nils-Göran Larsson, Arnaud Mourier

**Affiliations:** 1 Department of Mitochondrial Biology, Max Planck Institute for Biology of Ageing, Cologne, Germany; 2 Department of NanoBiophotonics, Max Planck Institute for Biophysical Chemistry, Göttingen, Germany; 3 Institute of Integrative Biology of the Cell (I2BC) UMR9198, CEA, CNRS, Univ. Paris-Sud, Université Paris-Saclay, Gif-sur-Yvette, France; 4 Computational Systems Biochemistry, Bioinformatics Core Facility, Max Planck Institute of Biochemistry, Martinsried, Germany; 5 INSERM U1163, Université Paris Descartes-Sorbonne Paris Cité, Institut Imagine, Paris, France; 6 Department of Laboratory Medicine, Karolinska Institutet, Stockholm, Sweden; 7 Aix-Marseille Université, CNRS, IBDM UMR 7288, Marseille, France; 8 Department of Medical Biochemistry and Biophysics, Karolinska Institutet, Stockholm, Sweden; 9 Université de Bordeaux, IBGC UMR 5095, Bordeaux, France; 10 CNRS, IBGC CNRS UMR 5095, Bordeaux, France; Stanford University School of Medicine, UNITED STATES

## Abstract

Mitochondrial dynamics is an essential physiological process controlling mitochondrial content mixing and mobility to ensure proper function and localization of mitochondria at intracellular sites of high-energy demand. Intriguingly, for yet unknown reasons, severe impairment of mitochondrial fusion drastically affects mtDNA copy number. To decipher the link between mitochondrial dynamics and mtDNA maintenance, we studied mouse embryonic fibroblasts (MEFs) and mouse cardiomyocytes with disruption of mitochondrial fusion. Super-resolution microscopy revealed that loss of outer mitochondrial membrane (OMM) fusion, but not inner mitochondrial membrane (IMM) fusion, leads to nucleoid clustering. Remarkably, fluorescence *in situ* hybridization (FISH), bromouridine labeling in MEFs and assessment of mitochondrial transcription in tissue homogenates revealed that abolished OMM fusion does not affect transcription. Furthermore, the profound mtDNA depletion in mouse hearts lacking OMM fusion is not caused by defective integrity or increased mutagenesis of mtDNA, but instead we show that mitochondrial fusion is necessary to maintain the stoichiometry of the protein components of the mtDNA replisome. OMM fusion is necessary for proliferating MEFs to recover from mtDNA depletion and for the marked increase of mtDNA copy number during postnatal heart development. Our findings thus link OMM fusion to replication and distribution of mtDNA.

## Author summary

Mammalian mitochondria contain multiple copies of the mitochondrial genome (mtDNA), which encodes genes that are essential for the oxidative phosphorylation system. An important feature of mtDNA is that it is evenly distributed throughout the mitochondrial network. Dynamin-related GTPase proteins help control the size and shape of mitochondria by fusion and fission events and are intimately linked to maintenance and distribution of mtDNA. Certain human mutations in mitofusin 2 (MFN2) and optic atrophy protein 1 (OPA1) cause disease phenotypes, such as peripheral neuropathy and optic atrophy, which are often also associated with mtDNA depletion. However, the mechanism whereby MFNs and OPA1 are involved in maintenance of mtDNA is unclear. In this study, we demonstrate that rapid mtDNA synthesis in proliferating tissue-culture cells or cardiomyocytes during post-natal heart development requires mitochondrial fusion. However, the absence of mitochondrial fusion in mouse heart is not associated with mtDNA integrity defects but instead affects the replication of mtDNA. These findings provide direct evidence for the importance of mitochondrial fusion in maintaining mtDNA replication.

## Introduction

Mammalian mitochondria are dynamic organelles present as long interconnected tubules or individual units that may undergo intracellular transport [1,2]. A family of dynamin-related GTPases regulate mitochondrial morphology through fission and fusion of the mitochondrial membranes [3–6]. The dynamin-related protein 1 (DRP1) mediates division of mitochondria, mitofusin 1 and 2 (MFN1 and MFN2) control outer mitochondrial membrane fusion, whereas optic atrophy 1 (OPA1) control inner mitochondrial membrane fusion. The above mentioned proteins are all essential for embryonic development [2,7,8], and mutated forms are known to cause human disease with phenotypes such as encephalopathy [9], peripheral neuropathy [10,11], and optic atrophy [12,13].

Mitochondrial dynamics is important for distribution and maintenance of mtDNA. Single mtDNA molecules are packaged by the DNA-binding mitochondrial transcription factor A (TFAM) into mitochondrial nucleoids that are evenly distributed throughout the mitochondrial network [14–16]. Previous research has reported that absence of mitochondrial fission results in an elongated mitochondrial network with bulbs harboring aggregates of mitochondrial nucleoids [17,18]. Additionally, inter-organellar contacts between the endoplasmic reticulum (ER) and the OMM have been proposed to facilitate the initial steps of mitochondrial division [19], and to be critical for mtDNA segregation after replication [20]. Thus, proteins acting on the OMM to promote mitochondrial division, possibly in coordination with ER contact sites, help distribute mitochondrial nucleoids. However, little is known about the role of mitochondrial fusion proteins in the distribution of nucleoids, in particular concerning mitofusins, which are also implicated in OMM-ER tethering.

Maintenance of mtDNA is absolutely dependent on mitochondrial fusion in budding yeast, whereas loss of fusion in mammals leads to depletion but not total loss of mtDNA [21–23]. The role for mitochondrial fusion in mtDNA maintenance is nicely illustrated by the double *Mfn1* and *Mfn2* heart conditional knockout animals (*dMfn* KO), where a strong reduction in mtDNA copy number impairs oxidative phosphorylation (OXPHOS) and cardiac function [23–25]. In skeletal muscle, the mtDNA depletion caused by absence of OMM fusion has been attributed to instability and increased mutagenesis of mtDNA [23]. Moreover, loss of IMM fusion through inducible OPA1 ablation in adult hearts leads to severe mtDNA depletion [26]. However, loss of mitochondrial fission induced by conditional knockout of DRP1 or reduced mitochondrial fusion due to conditional knockout of either MFN1 or MFN2 did not affect mtDNA copy number in cardiac tissue [23,27–29]. Fusion of the OMM and IMM, which serves to facilitate membrane and/or matrix content mixing, is thus critical for mtDNA copy number maintenance.

In this study, we generated *dMfn* KO conditional heart knockout mice and also characterized fusion-deficient mouse embryonic fibroblasts to decipher how nucleoid distribution and mtDNA maintenance are linked to mitochondrial fusion. Using sequencing of DNA, we found no increase in mtDNA rearrangements or point mutations in *dMfn* heart KO animals. Therefore, the low levels of mtDNA observed in the absence of OMM fusion are unlikely to be explained by increased mtDNA mutagenesis leading to instability. Additionally, we reveal that loss of OMM fusion leads to clustering of mitochondrial nucleoids, but this does not impair mitochondrial transcription. Notably, loss of OMM or IMM fusion alters the replisome protein composition, which impairs mtDNA replication activity. Altogether, we found that mitochondrial fusion is necessary to sustain high rates of mtDNA replication, but it is dispensable for maintaining integrity and transcription of mtDNA.

## Results

### Loss of OMM fusion leads to impaired mtDNA maintenance and OXPHOS deficiency

**Fig 1:**
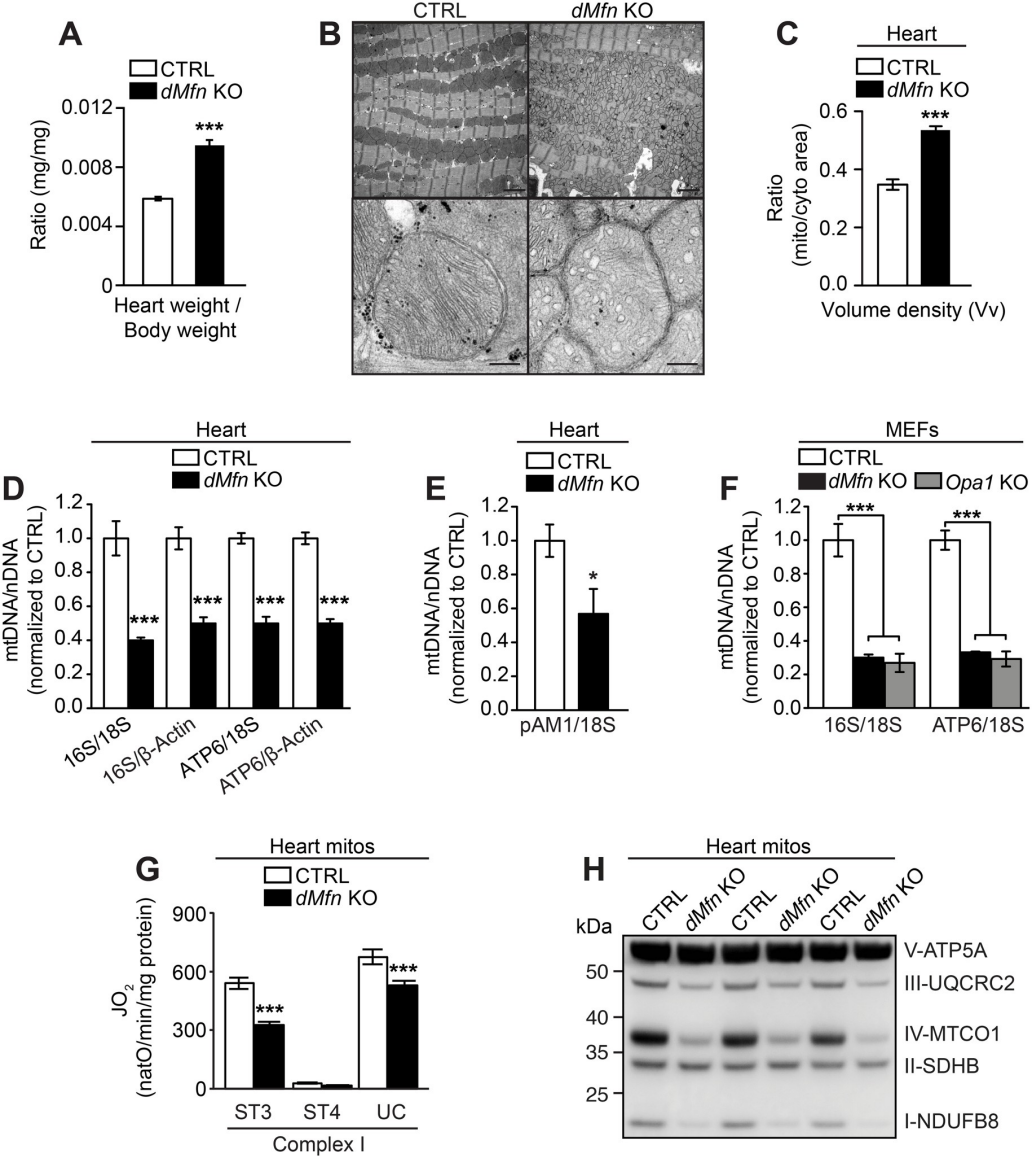
Loss of outer membrane fusion results in mtDNA depletion and OXPHOS dysfunction. (A) Heart weight to body weight ratio of male and female control (n = 19) and *dMfn* KO (n = 13) mice at 5 weeks of age. (B) Representative images of fixed heart tissue from control and *dMfn* KO animals at 5 weeks of age analyzed by electron microscopy. For each genotype 3–5 biological replicates were analyzed. Scale bars represent 2 μm for overview image and 0.2 μm for zoom-in image. (C) Volume density (Vv) analysis in heart of control (n = 5) and *dMfn* KO (n = 3) animals at 5 weeks of age as determined by ratio of mitochondria to cytoplasmic area from electron microscopy images. (D) Quantitative PCR analysis of mtDNA copy number (16S and ATP6) normalized to nuclear DNA (β-Actin and 18S) from heart samples of control (white bar) and *dMfn* KO (black bar) at 5 weeks of age (n = 5 of for each genotype). (E) Quantification of mtDNA levels by Southern blot analyses of control and *dMfn* heart KO samples (n = 3 for each genotype) at 5 weeks. The mtDNA levels (pAM1) were normalized to nuclear DNA (18S). (F) Quantitative PCR analysis of mtDNA copy number (16S and ATP6) normalized to nuclear DNA (β-Actin and 18S) from control (white bars), *dMfn* KO (black bars), *Opa1* KO (gray bars) MEFs (n = 4 of each genotype). (G) Oxygen consumption rates of heart mitochondria isolated from control and *dMfn* KO animals at 5 weeks of age, n = 10 for both genotypes. Mitochondrial respiration was assessed under phosphorylating (ST3), non-phosphorylating (ST4), and uncoupled (UC) conditions using complex I substrates. (H) Western blot of OXPHOS proteins from heart mitochondria from control and *dMfn* KO animals at 5 weeks of age (n = 3 for each genotype). Error bars indicate ± SEM. For (A, C, and G), Student T-test; *, P < 0.05; ***, P < 0.001. For (F), one-way ANOVA using Turkey’s multiple comparison test; ***, P < 0.001.

To study the link between mitochondrial fusion and mtDNA maintenance, we generated heart *dMfn* KO mice, which were born at the expected Mendelian ratios (S1A Fig) and showed loss of transcripts encoding MFN1 and MFN2 in heart tissue (S1B Fig). Most heart *dMfn* KO animals survived until the postnatal age of 5 weeks and displayed a significant increase in the heart weight to body weight ratio (Fig 1A). Transmission electron microscopy analysis of heart tissue revealed that *dMfn* KO mice exhibited a significant increase in ratio of mitochondrial/cytoplasmic area associated with aberrant mitochondrial ultrastructure and disruption of the myofibril organization (Fig 1B and 1C). In line with previous fusion-deficient models, qPCR and Southern blot analysis revealed a marked reduction of the mtDNA copy number in heart tissue from *dMfn* KO animals (Fig 1D and 1E) and in fusion-deficient *dMfn* KO and *Opa1* KO mouse embryonic fibroblasts (Fig 1F). High-resolution respirometry showed a significant decrease of respiration under phosphorylating and uncoupled conditions in *dMfn* KO heart mitochondria (Fig 1G) and in OMM and IMM fusion-deficient MEFs (S1C Fig), consistent with the observed mtDNA depletion (Fig 1D–1F). This respiratory defect correlated with a reduction in OXPHOS proteins in *dMfn* KO heart mitochondria (Fig 1H), which rely on mtDNA expression for their stability [30], and caused growth impairment of fusion-deficient MEFs (S1D Fig).

### Loss of mitochondrial fusion in heart does not affect mtDNA integrity

**Fig 2:**
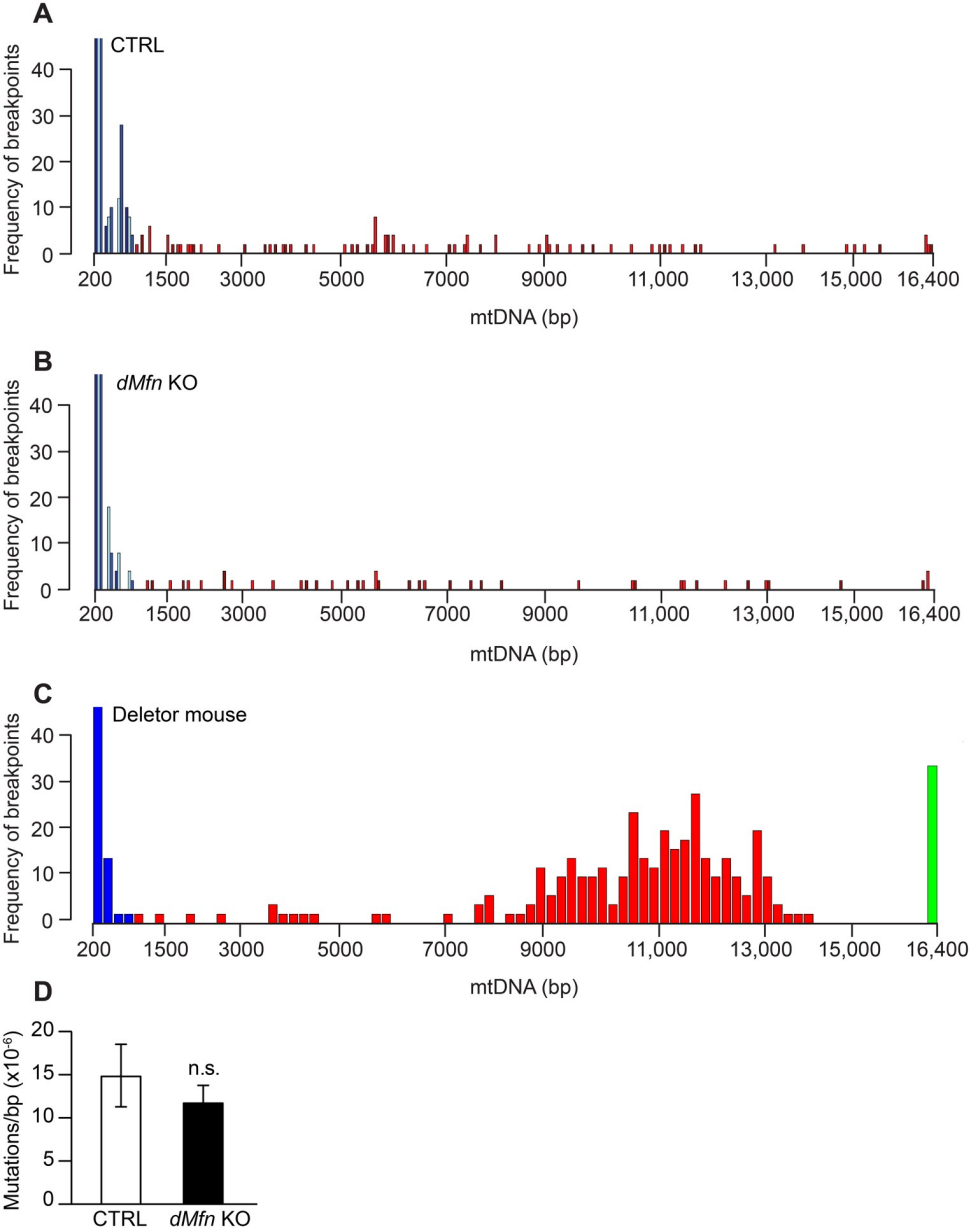
Integrity of mtDNA is unaffected upon loss of mitochondrial fusion. Analysis of mitochondrial DNA rearrangement frequency from hearts of control (A), *dMfn* KO (B), and Deletor (C) animals at 5 weeks of age as determined by Illumina sequencing (control n = 3; *dMfn* KO n = 3 and Deletor mouse n = 1). The graphs depict paired-end sequencing reads from individual animals with an abnormally long or short insert size plotted as bins along the x-axis. The frequency of unique rearrangement breakpoints is plotted on the y-axis. Blue bins represent variations in the fragment size and not rearrangement breakpoints; red bins denote rearrangements larger than 600 bases. In the deletor mice, a green bin at position 16,400 kb signifies rearrangement breakpoints encompassing the first and last positions of the reference sequence, leading to difficulties in assessing the true size of the rearrangement. Shades of blue and red indicate different individual animals within a group. (D) Mitochondrial DNA mutation load analysis in heart from control and *dMfn* KO animals at 5–6 weeks of age, n = 3 for both genotypes. n.s., no statistical significance based on Student T-test.

It has been shown that the absence of MFN1 and MFN2 in skeletal muscle severely reduces mtDNA copy number and leads to mtDNA integrity defects [23]. This observation has led to the prevailing theory stating that mitochondrial fusion safeguards mtDNA integrity and prevents mtDNA mutations [23,31]. To examine the integrity of mtDNA in heart tissue in the absence of OMM fusion, we performed paired-end Illumina sequencing to detect unique breakpoints consistent with rearrangements of mtDNA. Similar frequencies of mtDNA rearrangements were found in both negative control samples and *dMfn* KO hearts (Fig 2A and 2B). The positive control sample, which is derived from muscle tissue of a mouse that harbors a mutation in the DNA helicase Twinkle (A360T), which is known to cause mtDNA rearrangements in patients [32], contained abundant breakpoints consistent with mtDNA rearrangements (Fig 2C). Although this model has been described as a deletor mouse [32], the pattern of rearrangements we observed (Fig 2C) is consistent with abundant mtDNA duplications. Moreover, we found no difference in levels of mtDNA point mutations in heart tissue between control and *dMfn* KO animals by using post-PCR cloning and sequencing (Fig 2D). These results demonstrate that loss of OMM fusion in the heart reduces mtDNA copy number without influencing mtDNA integrity or increasing point mutations.

### Loss of OMM fusion affects nucleoid distribution

**Fig 3:**
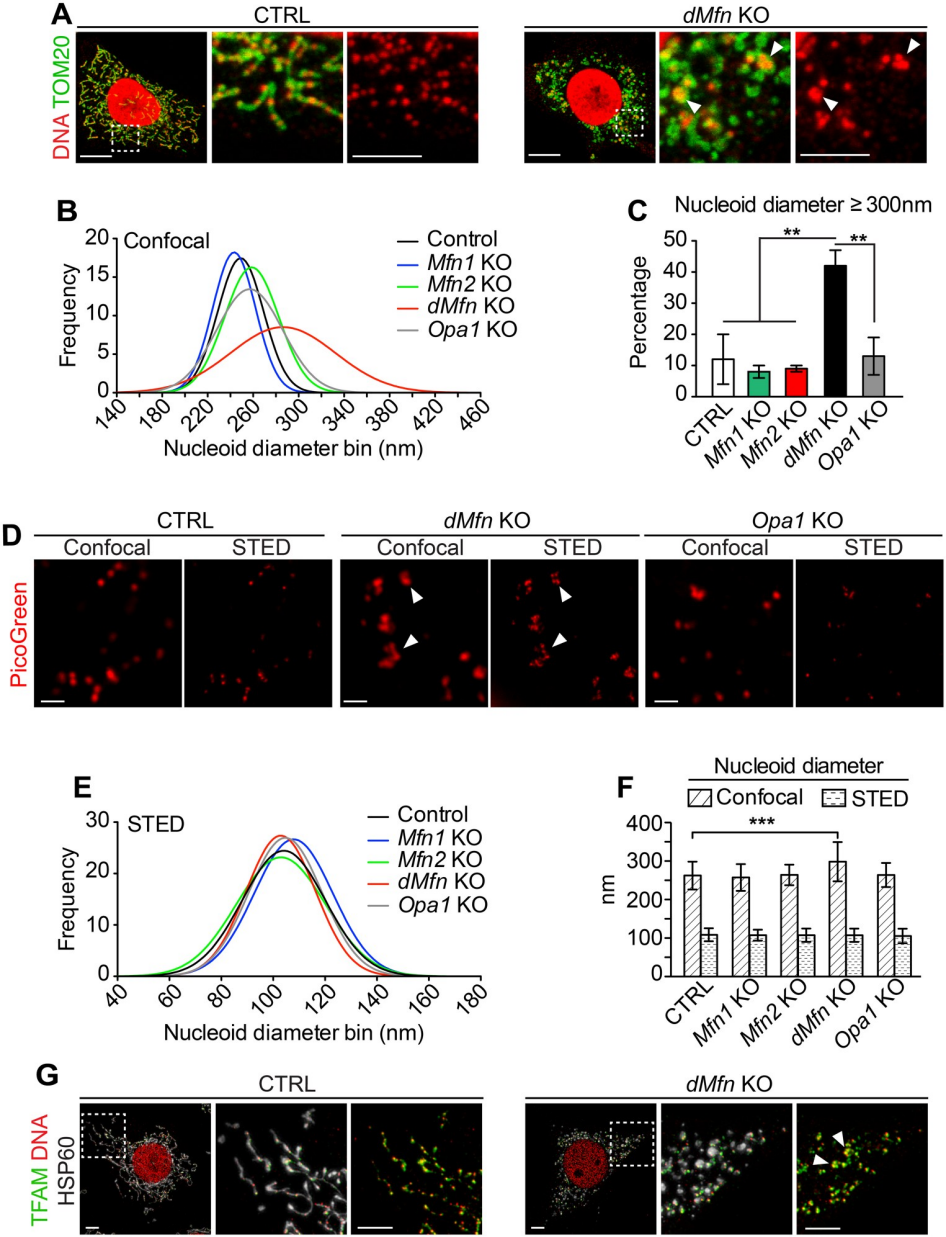
Loss of outer membrane fusion results in mitochondrial nucleoid clustering. (A) Confocal microscopy images of control and *dMfn* KO MEFs immunostained to detect TOM20 protein or DNA. Dashed boxes specify the areas of magnification shown in the panels to the right. Scale bars, main image 10 μm, zoom-in 5 μm. Three independent experiments were performed per genotype. (B) Gaussian distribution of diameters of PicoGreen-labeled nucleoids as determined by confocal microscopy of control, *Mfn1* KO, *Mfn2* KO, *dMfn* KO, and *Opa1* KO MEFs. (C) Quantification of PicoGreen-labeled nucleoids with a diameter ≥ 300 nm as determined by confocal microscopy. For each cell type (n = 3–6) up to 35 nucleoids were analyzed per cell. (D) Images of PicoGreen-labeled nucleoids from control, *dMfn* KO, *Opa1* KO MEFs. Scale bar 500 nm. (E) Gaussian distribution diameters of PicoGreen-labeled nucleoids from STED-acquired images in control, *Mfn1* KO, *Mfn2* KO, *dMfn* KO, *Opa1* KO MEFs. (F) Average diameters of PicoGreen-labeled nucleoids in control, *Mfn1* KO, *Mfn2* KO and *dMfn* KO MEFs from confocal and STED-acquired images. The nucleoid diameters were measured at full width at half maximum on 100 nucleoids from each genotype. Error bars indicate standard deviation of the mean. For each genotype, nucleoid diameters were determined from n = 3–6 cells. (G) Representative images from 3 independent experiments of control and *dMfn* KO MEFs stained with anti-TFAM, anti-DNA and anti-HSP60 antibodies. Scale bar is 5 μm. Error bars indicate ± SEM. For (C and F), one-way ANOVA using Turkey’s multiple comparison test; **, P < 0.01; ***, P < 0.001. Arrows indicate clustered mtDNA.

To investigate if the distribution of nucleoids throughout the mitochondrial network depends on mitochondrial fusion, we performed immunofluorescence staining against DNA and mitochondria in various fusion-deficient (*Mfn1* KO, *Mfn2* KO, *dMfn* KO, and *Opa1* KO) MEFs. Control MEFs immunostained with anti-DNA and anti-TOM20 antibodies, and imaged by confocal microscopy, showed abundant evenly distributed mitochondrial nucleoids throughout the mitochondrial network (Fig 3A). In contrast, *dMfn* KO MEFs displayed reduced abundance of nucleoids that often were present as enlarged foci in a subset of the enlarged-fragmented mitochondria (Fig 3A, white arrowheads; S2A and S2B Fig). The Gaussian distribution of confocal-determined nucleoid diameters revealed a high proportion of enlarged nucleoids in *dMfn* KO MEFs (Fig 3B), with ~40% of nucleoids having diameters ≥300 nm (Fig 3C). The abnormal size of nucleoids prompted us to further examine nucleoid morphology and diameter with stimulated emission depletion (STED) super-resolution microscopy [15]. In *dMfn* KO MEFs, the enlarged nucleoids observed by confocal microscopy were resolved into multiple nucleoids in very close proximity by STED microscopy (Fig 3D, white arrowheads). This finding was obtained by using either PicoGreen or anti-DNA antibody staining to determine nucleoid morphology (Fig 3D and S2C Fig). Control MEFs and fusion knockout MEFs of different genotypes all exhibited nucleoid diameters of about 100 nm when analyzed by STED microscopy (Fig 3E and 3F, and S2D and S2E Fig), in agreement with published reports [33,34]. These observations show that loss of OMM fusion in MEFs does not alter nucleoid size but instead causes nucleoids to cluster. We also visualized nucleoids by confocal microscopy using antibodies against TFAM, which is the core protein packaging mtDNA into nucleoids, together with antibodies against DNA. As expected, we observed that TFAM is present in every nucleoid in both control and *dMfn* KO MEFs (Fig 3G). The morphology of nucleoids was similar when visualized with TFAM or DNA antibodies. Notably, the stoichiometry between TFAM and mtDNA was maintained among the nucleoid population, as clustered nucleoids exhibit more TFAM reactivity than single nucleoids. This is in line with our finding that the enlarged nucleoids are composed of multiple nucleoids, and thus exhibit greater TFAM reactivity. This observation suggests that insufficient compaction by TFAM cannot account for the enlarged nucleoids observed by confocal microscopy.

**Fig 4:**
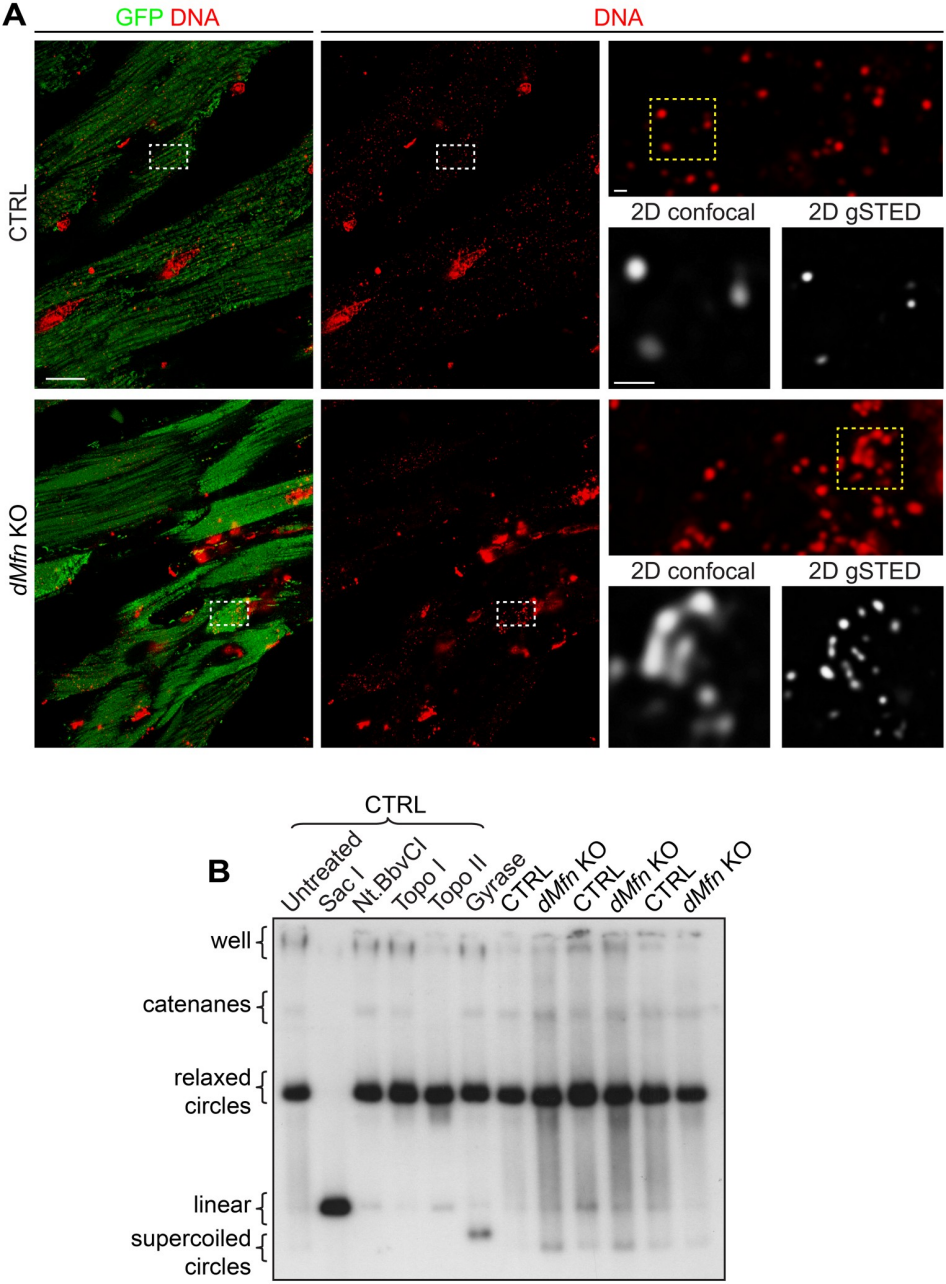
Loss of outer membrane fusion does not affect mtDNA topology. (A) Representative deconvoluted confocal and STED images of mtDNA from heart sections of control and *dMfn* KO animals at 4 weeks of age. Tissue samples were stained with anti-GFP and anti-DNA antibodies. Left panels show maximal projection of heart sections for mitochondria (GFP), nuclear and mitochondrial DNA (red). White dashed boxes specify areas of magnification shown in top right panel. Bottom right panels show 2D (single stack) images of mitochondrial nucleoids obtained by dual confocal and gated STED (gSTED) microscopy corresponding to the yellow dashed boxed. Scale bars represent 10 μm for overview image, and 500 nm in both zoom-in panels. Staining patterns were determined in three independent animals per genotype. (B) Topology of mtDNA obtained by using total DNA from heart tissue of control (n = 3) and *dMfn* KO (n = 3) mice at 3 weeks of age. The high molecular weight portion of the gel is shown. Control mtDNA was either untreated or treated with SacI, NtBbvCI, topoisomerase I (Topo I), topoisomerase II (Topo II), or DNA gyrase enzymes. mtDNA was probed with pAM1 (mouse mtDNA).

To rule out the possibility that the observed nucleoid clustering phenotype could be restricted to cultured MEFs, we investigated nucleoid appearance *in vivo*. The mitochondrial network and nucleoids were immunostained in heart tissue sections from control and conditional *dMfn* KO animals. In line with the results from MEFs, confocal imaging of *dMfn* KO heart sections confirmed the presence of apparently large nucleoids that were resolved into multiple nucleoids by STED microscopy (Fig 4A). Furthermore, the different topological conformations of mtDNA in heart tissue were examined and we found no difference in the amount of mtDNA catenanes, which are physically interlocked molecules (Fig 4B). Therefore, changes in mtDNA topology could not explain the clustering of nucleoids in *dMfn* KOs. Altogether, our analyses reveal that OMM fusion does not influence nucleoid size, but it is required for proper nucleoid distribution in cultured cells and *in vivo*.

### Mitochondrial fusion is dispensable for mitochondrial transcription

**Fig 5:**
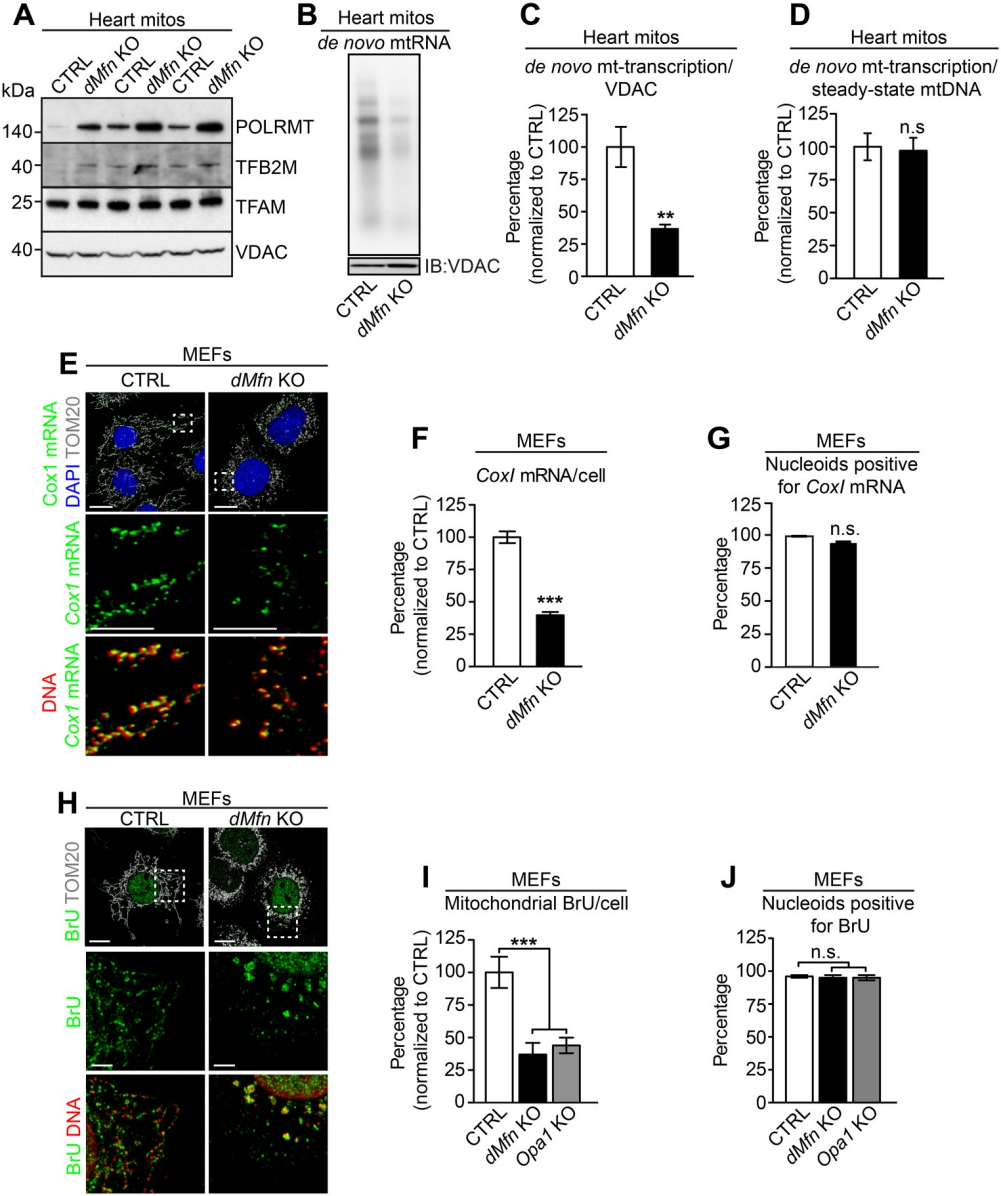
Loss of mitochondrial fusion does not affect mtDNA transcription. (A) Western blot analysis of steady-state levels of proteins essential for mitochondrial transcription in heart mitochondria from control and *dMfn* heart KO animals at 5–6 weeks of age (n = 3 for each genotype). (B) Representative image of a mitochondrial *de novo* transcription assay with heart mitochondria. Mitochondrial VDAC levels were determined by western blot analysis and used as loading control. Multiple control (n = 6) and *dMfn* KO (n = 5) heart mitochondrial samples were analyzed at 4 weeks of age. (C) Quantification of mitochondrial *de novo* transcription related to VDAC protein levels in control (n = 6) and *dMfn* heart KO (n = 5) mitochondria. (D) Quantification of mitochondrial *de novo* transcription related to the steady-state levels of mtDNA of control (n = 6) and *dMfn* KO (n = 5) heart mitochondria. (E) Representative fluorescence in situ hybridization of images visualizing the mitochondrial *CoxI* mRNA, followed by immunocytochemistry to detect mtDNA and TOM20 protein in control and *dMfn* KO MEFs. Dashed boxes specify the areas of magnification. Scale bars represent 10 μm for overview image and 5 μm for zoom-in images. Staining patterns were determined in five independent experiments per genotype. (F) Quantification of *CoxI* mRNA in control (n = 17) and *dMfn* KO (n = 15) by using stacked confocal images from individual MEFs. (G) Quantification of nucleoids positive for *CoxI* mRNA by using stacked confocal images from control (n = 15) and *dMfn* KO (n = 14) MEFs. (H) Representative images of immunocytochemistry to detect BrU labeling of newly synthesized mitochondrial RNA, mtDNA and TOM20 protein in control and *dMfn* KO MEFs. Dashed boxes specify the areas of magnification. Scale bars represent 10 μm for overview image and 5 μm for zoom-in images. Staining patterns were determined in five independent experiments per genotype. (I) Quantification of BrU-labeled mitochondrial RNAs by using stacked confocal images from control (n = 15), *dMfn* KO (n = 15), and *Opa1* KO (n = 15) MEFs. (J) Quantification of BrU-positive nucleoids by using stacked confocal images from control (n = 15), *dMfn* KO (n = 18), and *Opa1* KO (n = 15) MEFs. Error bars indicate ± SEM. For (C, D, F, and G), Student T-test; **, P < 0.01; ***, P < 0.001. For (I and J), one-way ANOVA using Turkey’s multiple comparison test relative to control; ***, P < 0.001; n.s., non-significant difference.

To determine how mitochondrial fusion impacts mtDNA copy number, we first evaluated mitochondrial transcription as the mitochondrial RNA polymerase (POLRMT) provides the RNA primers required for initiation of mtDNA replication [35]. There was a general reduction of steady-state levels of mitochondrial transcripts in hearts of conditional *dMfn* KO animals, whereas the positive (*Mterf4* KO) and negative (*Polrmt* KO) control samples showed the expected increase and decrease in transcripts, respectively (S3A Fig). Also, levels of the promoter-proximal 7S RNA, previously shown to correlate with transcription initiation [35], were reduced in heart *dMfn* KO animals (S3B Fig). The reduction of mitochondrial transcript levels including 7S RNA was proportional to the reduction of mtDNA levels (Fig 1D). Furthermore, the levels of the key mitochondrial transcription factors TFAM and mitochondrial transcription factor B2 (TFB2M) were normal, whereas POLRMT levels were increased (Fig 5A). These findings suggest that the reduced abundance of mtDNA templates for transcription explains the reduced levels of mitochondrial transcripts. To investigate this possibility further, we performed *in organello* transcription assays on isolated heart mitochondria and observed an impaired mitochondrial transcription in *dMfn* KO heart mitochondria (Fig 5B and 5C). However, there was no difference between control and *dMfn* KO heart mitochondria when levels of *de novo* transcripts were normalized to the levels of mtDNA templates (Fig 5D). We proceeded to assess transcription in individual nucleoids by applying FISH to detect the mtDNA-encoded *CoxI* mRNA in MEFs (Fig 5E). The levels of *CoxI* mRNA as determined by FISH were reduced in *dMfn* KO MEFs (Fig 5F), consistent with the northern blot results (S3A Fig). Importantly, the *CoxI* mRNA was detected in close proximity to almost all nucleoids in both control and *dMfn* KO MEFs (Fig 5E and 5G) showing that nucleoid clustering does not alter transcription activity. As the FISH analyses of *CoxI* mRNA does not directly reflect ongoing mtDNA transcription, we also performed bromouridine (BrU) labeling to assess *de novo* transcription in MEFs. In agreement with the results from FISH analysis of the *CoxI* mRNA, we found that mitochondrial BrU incorporation was decreased by more than 50% in *dMfn* and *Opa1* KO MEFs (Fig 5H and 5I). Notably, the vast majority of nucleoids in control as well as in OMM or IMM fusion-deficient MEFs incorporated BrU into newly synthesized transcripts (Fig 5H and 5J). These results show that mitochondrial transcription is proportional to the number of nucleoids, i.e. the abundance of mtDNA templates, and that clustering of nucleoids does not impair mtDNA transcription.

### Loss of mitochondrial fusion causes severe imbalance of replisome proteins

The finding of preserved transcription in *dMfn* KO animals (Fig 5) makes it unlikely that RNA primer formation limits mtDNA replication. Normally, the vast majority of all initiated mtDNA replication events are prematurely terminated after about 650 nucleotides to generate a short single-stranded species denoted 7S DNA [36]. Southern blot (S3C Fig) and qPCR analyses (S3D Fig) consistently showed that the levels of 7S DNA normalized to mtDNA levels were unchanged in *dMfn* KO hearts. We also included control samples from *Mgme1* whole body KO and *Polrmt* heart KO animals that showed the expected increase and decrease of 7S DNA levels, respectively [35,37]. Our results thus suggest that initiation of mtDNA replication is unaffected in OMM fusion-deficient hearts and the mtDNA depletion must therefore be caused by events downstream of initiation.

**Fig 6:**
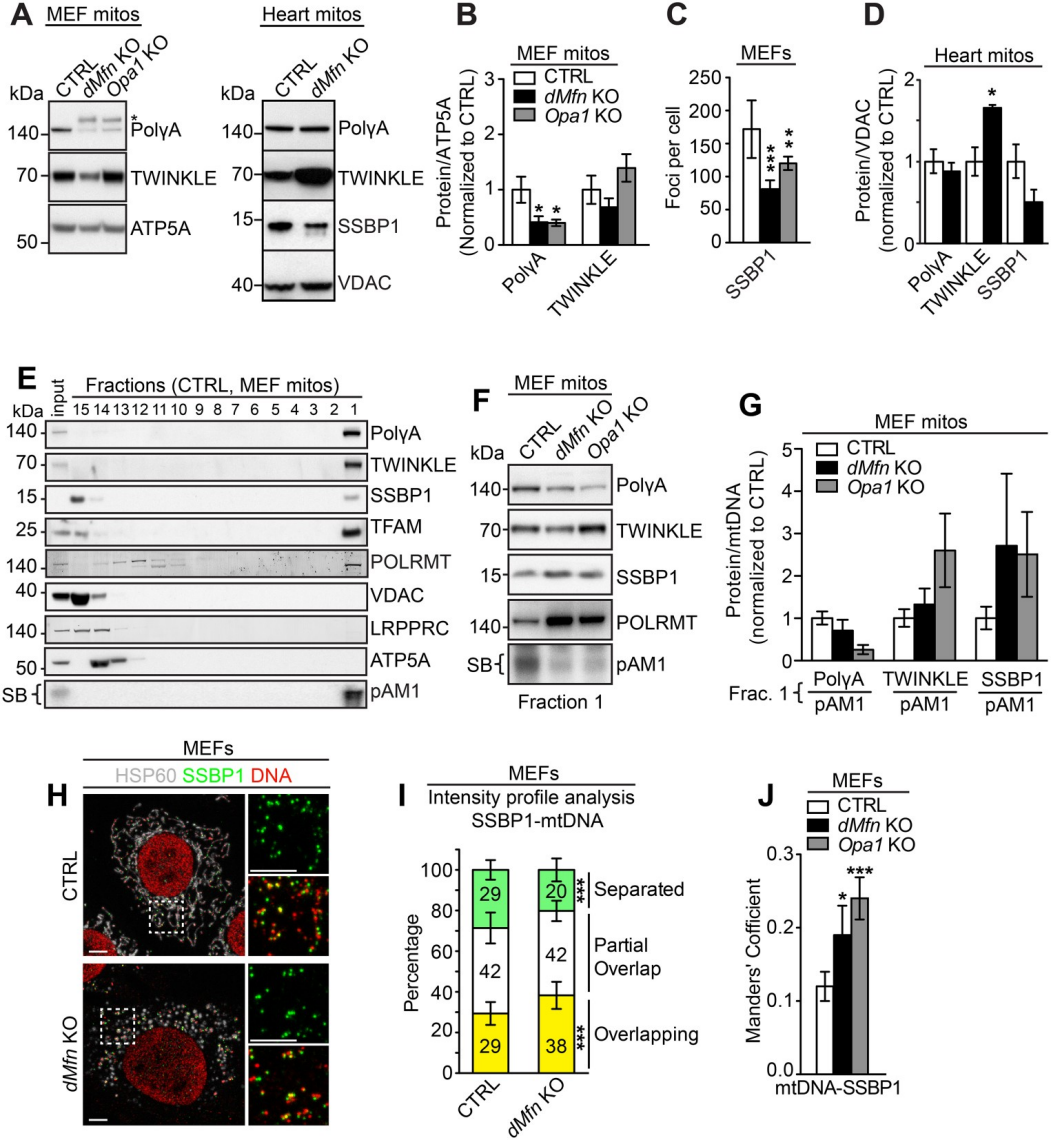
Loss of mitochondrial fusion affects replisome composition. (A) Left, representative western blot showing the steady-state levels of mitochondrial replication proteins from isolated MEF mitochondria from control, *dMfn* KO, *Opa1* KO MEFs. Right, representative western blot showing the steady-state levels of mitochondrial replication proteins in isolated heart mitochondria from control and *dMfn* KO mice at 5–6 weeks of age. (B) Quantification of the steady-state levels of replisome proteins as determined by western blot analysis of mitochondria from control, *dMfn* KO, and *Opa1* KO MEFs. Replisome protein levels were normalized to ATP5A (n = 7 per genotype). (C) Quantification of SSBP1 foci per cell in control, *dMfn* KO, and *Opa1* KO MEFs. Cells were immunostained with anti-SSBP1 and anti-HSP60 antibodies, n = 4 for each genotype, 5–8 cells analyzed per n. (D) Quantification of mitochondrial replication proteins levels in isolated heart mitochondria from control (n = 3) and *dMfn* KO (n = 3) mice at 5–6 weeks of age, normalized to VDAC. (E) Representative western blot analysis of glycerol gradient fractions from mitochondria isolated from control MEFs. Upper panel corresponds to western blotting and lower panel shows Southern blot (SB) analysis to detect mtDNA by using the pAM1 probe. (F) Representative western blot analysis of fraction 1 from the glycerol density gradient. In total, 4 independent biological samples were used for all genotypes. (G) Quantification of the western blot analysis of fraction 1 from the glycerol density gradient related to (F), n = 4 for all genotypes. (H) Representative confocal image of control and *dMfn* KO MEFs immunostained with anti-SSBP1 and anti-dsDNA antibodies, n = 3 for each genotype. Scale bars represent 5 μm. (I) Line scan analysis based on intensity profiles of SSBP1 and mtDNA of control and *dMfn* KO MEFs, n = 3 for each genotype, 7–10 cells analyzed per n. Line scans generated intensity profiles that were separated into three categories, no overlap of intensities between SSBP1-mtDNA (free SSBP1), partial overlap between SSBP1-mtDNA intensities, and complete overlap of intensities between SSBP1-mtDNA. (J) Mander’s Coefficient, related to H, expressing the degree of mtDNA foci colocalizing with SSBP1 foci from confocal acquired images in control (n = 4 independent experiments, 23 cells analyzed per n) and *dMfn* KO (n = 3 independent experiments, 16 cells analyzed per n), *Opa1* KO MEFs (n = 3 independent experiments, 22 cells analyzed per n). For all, error bars indicate ± SEM. (For D and I) Student T-test; *, P < 0.05; **, P < 0.01; ***, P < 0.001. For (B, C, G, and J), one-way ANOVA using Turkey’s multiple comparison test relative to control; *, P < 0.05; **, P < 0.01; ***, P < 0.001.

The basal mitochondrial replisome is composed of the mitochondrial DNA polymerase γ (POLγ), which consists of the catatytic (POLγA) and the accessory (POLγB) subunits. In addition, the DNA helicase TWINKLE and the single-stranded DNA binding protein 1 (SSPB1) are components of the basal replisome. Western-blot analyses of POLγA, TWINKLE, and SSBP1 levels revealed a striking imbalance between the steady-state levels of replisome components in *dMfn* KO hearts and MEFs (Fig 6A). Low levels of SSBP1 were consistently observed in both *dMfn* KO hearts (Fig 6A and 6D) and MEFs (Fig 6C). TWINKLE levels were normal in mutant MEFs but upregulated in *dMfn* KO hearts. In contrast, the PolγA levels were drastically reduced in IMM and OMM fusion incompetent MEFs and near normal in *dMfn* KO hearts (Fig 6A–6D). Interestingly, the severity of replisome imbalance observed between fusion incompetent mitochondria in heart tissue and MEFs (Fig 6A–6D) nicely correlate with the extent of mtDNA depletion observed between these two models (Fig 1D–1F). To further define the nucleoid protein composition, we subjected Triton-X-lysed MEF mitochondria to density gradient centrifugation to enrich for nucleoid-associated proteins under native conditions. We found a lower ratio of POLγA per mtDNA in the nucleoid-enriched fraction in both *dMfn* and *Opa1* KO MEFs (Fig 6E–1G). Notably, all the fusion-deficient mitochondria analyzed by density gradients consistently showed an altered stoichiometry of mitochondrial replisome factors associated with the mtDNA template (Fig 6F and 6G). In support of this finding, image analysis showed that SSBP1 co-localized to a greater extent with nucleoids in IMM and OMM incompetent MEFs compared to nucleoids in control MEFs (Fig 6H–6J). Mitochondrial DNA replication not only requires a functional replisome but also relies on stable supply of deoxyribonucleotides (dNTPs). To rule out changes in the cellular dNTP pools as a causative reason for mtDNA depletion in fusion-deficient MEFs, we determined the abundance of dNTPs by UPLC-MS and found no difference between control, *dMfn KO*, and *Opa1* KO MEFs (S4A Fig).

### OMM fusion is necessary to sustain high rates of mtDNA replication during postnatal heart development

**Fig 7:**
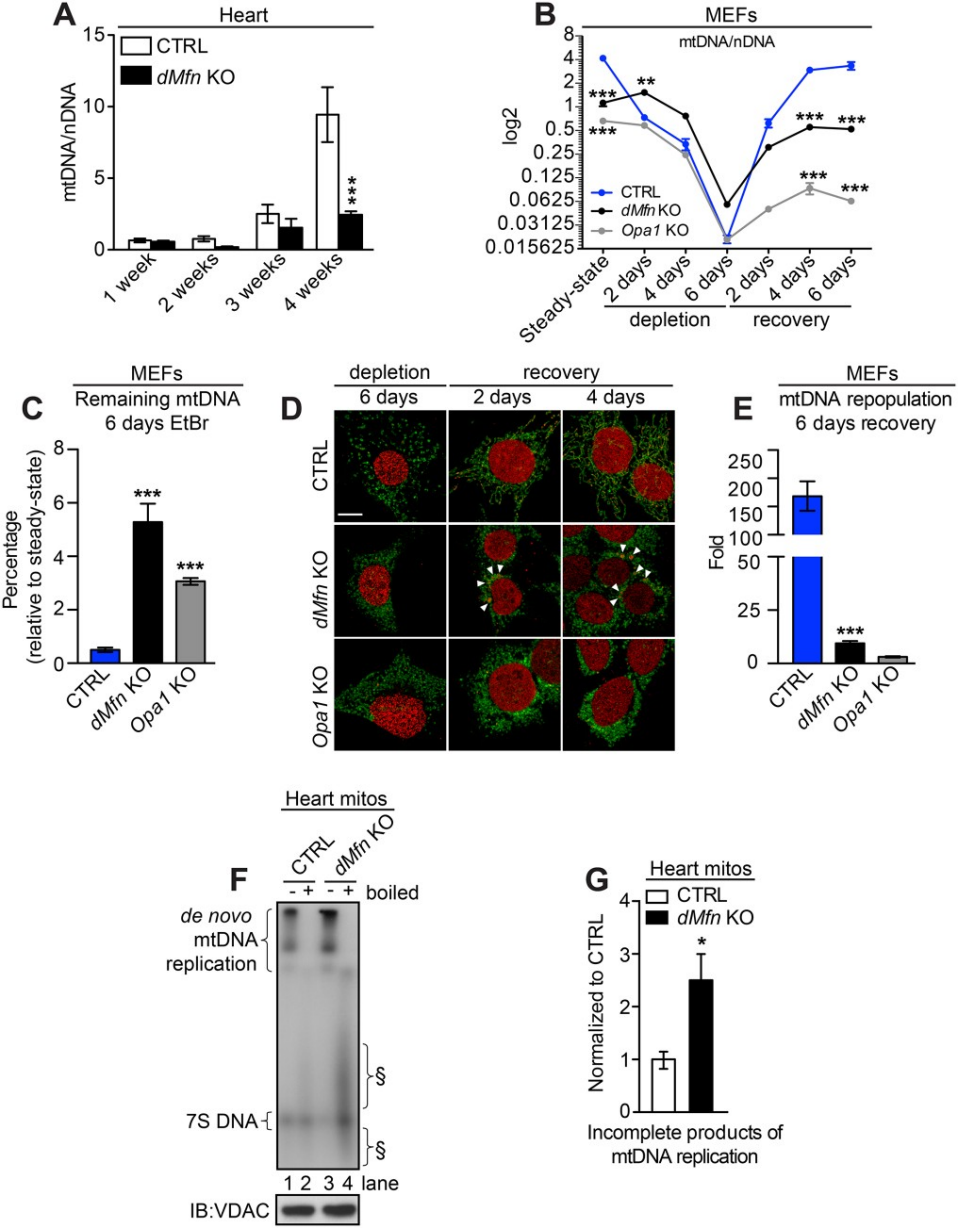
Loss of mitochondrial fusion impairs mtDNA replication. (A) Quantitative PCR analysis of mtDNA copy number (ATP6) normalized to nuclear DNA (18S) in heart samples from control and *dMfn* KO between 1–4 weeks of age (n = 3–10 per genotype). (B) Quantitative PCR analysis of mtDNA copy number (ATP6) normalized to nuclear DNA (18S) from control, *dMfn* KO, and *Opa1* KO MEFs after treatment with 100 ng/ml ethidium bromide (EtBr), n = 4–8 replicates per genotype. (C) Quantification of mtDNA levels in control, *dMfn* KO, and *Opa1* KO MEFs after 6 days of treatment with 100 ng/ml EtBr, n = 4 for all genotypes. (D) Representative confocal images of control, *dMfn* KO, and *Opa1* KO MEFs during and after EtBr treatment. Cells were immunostained with anti-dsDNA and anti-TOM20 antibodies, n = 3 for each genotype. Scale bars represent 10 μm. White arrows point to clustered nucleoids. (E) Quantification of the fold change of mtDNA after 6 days of recovery from treatment with 100 ng/ml EtBr in control, *dMfn* KO, and *Opa1* KO MEFs (n = 4 for all genotypes). (F) Mitochondrial *de novo* replication assay in heart mitochondria from control (n = 12) and *dMfn* KO (n = 8) animals at 3–4 weeks of age, normalized to VDAC protein levels (bottom panel). Incomplete mtDNA replication products (§) are indicated. Numbers mark the different mtDNA lanes (G) Quantification of the abundance of incomplete mtDNA replication products produced *de novo* in control (n = 6) and *dMfn* KO (n = 7) heart mitochondria at 3–4 weeks of age. For all, error bars indicate ± SEM. (A) and (B), two-way ANOVA using Bonferroni multiple comparison test; **, P < 0.01; ***, P < 0.001. (C) and (D), one-way ANOVA using Turkey’s multiple comparison test; ***, P < 0.001. (G) Student T-test; *, P < 0.05.

The postnatal development of cardiomyocytes involves substantial mtDNA replication leading to an almost 13-fold increase of mtDNA copy number during the first four weeks of postnatal life (Fig 7A). Interestingly, the *dMfn* KO hearts cannot sustain this increase and develop severe mtDNA depletion at age 4 weeks (Fig 7A). The low steady-state levels of mtDNA show that loss of OMM fusion in heart tissue prevents the mtDNA increase normally occurring during postnatal heart development. To investigate whether the replisome protein imbalance directly impacts mtDNA replication in cells lacking mitochondrial fusion, we treated MEFs with ethidium bromide (EtBr) and studied mtDNA depletion and repletion dynamics. We observed that the rate of mtDNA depletion was faster in control MEFs than in *dMfn* or *Opa1* KO MEFs (Fig 7B and 7C), suggesting that the rate of mtDNA loss induced by EtBr may depend on mtDNA replication rates. Notably, after six days of EtBr treatment, clustered nucleoids were no longer detected in *dMfn* KO MEFs (Fig 7D). However, during the repopulation phase, clustered nucleoids reemerged extensively in *dMfn* KO MEFs (Fig 7D). Moreover, during the mtDNA repopulation phase, MEFs with loss of IMM or OMM fusion showed a severe decrease in the mtDNA synthesis rate (Fig 7B and 7E). To further study the mtDNA replication process, we performed *in organello* assays with *dMfn* KO heart mitochondria and followed the incorporation of a radioactively labeled nucleotide into newly synthesized mtDNA. A marked increase in incomplete mtDNA replication products, smaller than full-length mtDNA, was visible as smears surrounding the 7S DNA in *dMfn* KO heart mitochondria (Fig 7F and 7G). We have previously observed that the formation of similar incomplete mtDNA replication products are linked to mtDNA replication stalling and mtDNA depletion in *Mgme1* KO animals [37]. It is thus plausible that *dMfn* KOs exhibit processivity defects during mtDNA replication in the absence of mitochondrial fusion. Altogether, our analyses reveal that mitochondrial fusion is necessary to maintain high replicative activity of mtDNA.

## Discussion

The mechanism whereby loss of OMM fusion leads to mtDNA depletion is unclear and a popular theory has been that fusion stabilizes mtDNA by preventing mutagenesis and instability. However, this hypothesis, based on the observation that skeletal muscle *dMfn* KO mice accumulate point mutations and deletions of mtDNA [23], is unsatisfactory because the absolute levels of point mutations and deletions were extremely low (2-3x10^-6^ mutations/bp and 1x10^-6^ deletions/mtDNA). If one assumes ~10^3^ mtDNA copies per nucleus in skeletal muscle cells, then only 1:10^3^ of the nuclear domains in skeletal muscle will contain a deletion or a point mutation of mtDNA. Contrary to this previous model, we show here that heart *dMfn* KO mice have no increase of point mutations or deletions of mtDNA, which excludes this mechanism as a cause for mtDNA depletion. Importantly, our results are based on loss-of-function mutations and it should be pointed out that missense mutations in MFN2 or OPA1 in humans have been reported to cause mtDNA deletions, whereas, in support of our findings, frameshift or premature stop mutations that lead to loss of the fusion protein, do not seem to cause mtDNA deletions in humans [38,39].

Characterization of heart *dMfn* KO animals revealed that loss of OMM fusion causes a severe imbalance of replisome factors with mtDNA depletion as a direct consequence. However, in contrast to MEFs, TWINKLE protein levels in *dMfn* KO hearts are strongly upregulated, consistent with the findings in other heart conditional KO mice with mtDNA replication deficiencies. The increase of TWINKLE in KO hearts may represent a compensatory response to counteract mtDNA depletion [35]. The lack of a similar response in KO MEFs could explain why the replisome factor imbalance and mtDNA depletion are more severe in fibroblasts than in heart tissue. Altogether, our results show that both OMM and IMM fusion ensure rapid rates of mtDNA replication. This may be achieved by equilibrating the stoichiometry of replisome factors in order to allow the formation of functional replisomes throughout the mitochondrial network. This is well in line with previous *in vitro* studies that have shown that a balanced replisome composition is essential for efficient mtDNA replication [40–43]. Consistent with these data, mtDNA depletion phenotypes have also been reported *in vivo* when replisome proteins have been overexpressed or knocked-out in animals [44–49]. The results we present here thus suggest that mitochondrial content mixing induced by mitochondrial dynamics is necessary to maintain the delicate protein composition balance of the mitochondrial replisome.

Our study also shows that OMM fusion is an important player that determines segregation of mitochondrial nucleoids. The lack of nucleoid distribution was not strictly related to a general loss of fusion, because *Opa1* KO mitochondria did not display nucleoid clustering. Instead, this phenomenon appears to be specific to cells with a combined loss of both MFN1 and MFN2. It has previously been reported that distribution of mitochondrial nucleoids depends on fission induced by DRP1 and that mtDNA replication preferentially may occur at ER-OMM contact sites [17,20,50]. It is thus possible that the OMM serves as an important platform for mtDNA distribution and that mitofusins are involved in this process. In line with this hypothesis, clustered nucleoids were no longer visible in *dMfn* KO MEFs during EtBr treatment but reappeared during the mtDNA repopulation phase. This suggests that nucleoid clusters are not caused by altered mtDNA stability, but rather result from impaired mtDNA distribution when OMM fusion is absent. Future studies are warranted to further understand the molecular link between nucleoid distribution and OMM dynamics. In summary, we report here an unexpected link between mitochondrial fusion and mammalian mtDNA replication, whereby mitochondrial fusion coordinates high rates of mtDNA replication and promotes mitochondrial nucleoid distribution.

## Material and methods

### Contact for reagent and resource sharing

Further information and requests for resources and reagents should be directed to and will be fulfilled by the lead contact, Nils-Göran Larsson (nils-goran.larsson@ki.se)

### Ethics statement

All animal procedures were conducted in accordance with European, national and institutional guidelines and protocols were approved by the Landesamt für Natur, Umwelt und Verbraucherschutz, Nordrhein-Westfalen, Germany. Animal work also followed the guidelines of the Federation of European Laboratory Animal Science Associations (FELASA).

### Mice

C57BL/6N mice with loxP-flanked *Mfn1* and *Mfn2* genes were previously described in Lee *et al*., 2012 [51]. To generate *Mfn1* and *2* heart conditional double knockout mice, male mice homozygous for mitofusin 1, heterozygous for mitofusin 2, and heterozygous for expression of cre-recombinase in heart and skeletal muscle (*Mfn1*^loxP/loxP^, *Mfn2*^*+/l*oxP^; Ckmm-cre^-/+^), were crossed with female mice homozygous for loxP-flanked mitofusin 1 and 2 genes (*Mfn1*^loxP/loxP^, *Mfn2*^*loxP/l*oxP^). Littermates lacking the transgenic Cre-recombinase allele were used as controls. In some crosses, an allele allowing for the ubiquitous expression of a mitochondria-targeted YFP from the ROSA26 locus was also introduced.

### Cell lines

Immortalized MEFs with homozygous knockout for *Mfn1*, *Mfn2* or both *Mfn1* and *Mfn2*, were originally generated in the lab of Dr. David Chan [2,52]. Mitofusin and *Opa1* KO MEFs were obtained from Dr. Thomas Langer. MEFs were maintained in DMEM GlutaMax containing 25 mM glucose (Thermo Fisher Scientific, 31966–021) supplemented with 10% fetal bovine serum (Thermo Fisher Scientific, 10270–106), 1% penicillin/streptomycin (Thermo Fisher Scientific, 15070–063), 1% non-essential amino acids (Thermo Fisher Scientific, 11140–050), and 50 μg/ml uridine. Cells were passaged every 3 to 4 days.

### Transmission electron microscopy

Electron micrographs of heart mitochondria from control and *dMfn* heart double knockout mice were obtained as previously described [53]. Small pieces from the left myocardium were fixed in a mix of 2% glutaraldehyde and 1% paraformaldehyde in 0.1 M phosphate buffer pH 7.4, at room temperature for 30 min, followed by 24 hours at 4°C. Specimens were rinsed in 0.1 M phosphate buffer and post-fixed in 2% OsO_4_ for 2 hours, dehydrated and embedded in LX-112 resin. Ultra-thin sections (approximately 50–60 nm) were cut and sections were examined in a transmission electron microscope (Tecnai 12, FEI Company, Netherlands) at 80 kV. Digital images at a final magnification of 8,200x were randomly taken on myofibrils from sections of the myocardium. The volume density (Vv) of mitochondria was calculated on printed digital images by point counting using a 2 cm square lattice [54]. To determine the number of images needed for an appropriate sampling, we used a cumulative mean plot [54]. In total, 15 randomly taken images were used from each animal.

### Mitochondria isolation

Mice were sacrificed by cervical dislocation and hearts were quickly washed in ice-cold PBS. Hearts were then minced and gently homogenized using a Potter S homogenizer (Sartorius) in 5 ml of mitochondria isolation buffer (MIB, 310 mM sucrose, 20 mM Tris-HCl, and 1 mM EGTA). Differential centrifugation of homogenates was performed to isolate intact mitochondria. First, homogenates were centrifuged for 10 min at 1,000x*g* at 4°C. Then, supernatants were collected and centrifuged again for 10 min at 4,500x*g* at 4°C. Crude mitochondria were resuspended in MIB and the protein concentration was determined by using the DC Protein assay (Bio-Rad).

### Mitochondrial respiration

The flux of mitochondrial oxygen consumption in isolated heart mitochondria was measured as previously described [55]. The assay was performed on 100–125 μg of crude heart mitochondria diluted in 2 ml of mitochondria respiration buffer (120 mM sucrose, 50 mM KCl, 20 mM Tris-HCl, 4 mM KH_2_PO_4_, 2 mM MgCl_2_, and 1 mM EGTA, pH 7.2) in an Oxygraph-2K (Oroboros Instruments) at 37°C. The mitochondria oxygen consumption rate was evaluated using either 10 mM pyruvate, 5 mM glutamate, and 5 mM malate. The oxygen consumption flux was assessed in the phosphorylating state with 1 mM ADP or in the non-phosphorylating state by addition of 2.5 μg/ml oligomycin. Lastly, mitochondrial respiration was uncoupled by successive addition of CCCP up to 3 μM to reach maximal oxygen consumption. The respiratory control ratio values were >10 with pyruvate/glutamate/malate and >5 with succinate/rotenone based on control heart mitochondria.

### Western blotting

Heart tissue and whole cells were lyzed in RIPA buffer (150 mM sodium chloride, 1.0% NP-40 or Triton X-100, 0.5% sodium deoxycholate, 0.1% SDS (sodium dodecyl sulfate), 50 mM Tris, pH 8.0). Isolated heart mitochondria were resuspended in NuPAGE LDS Sample Buffer (novex, NP0007) with 0,1M dithiothreitol, and proteins were separated by SDS-PAGE using 4–12% precast gels (Invitrogen), followed by blotting onto polyvinylidene difluoride (PVDF) membranes or in particular cases onto nitrocellulose membranes (GE Healthcare). Membranes were blocked in 3% milk/TBS. The following primary antibodies were used: mouse anti-OXPHOS cocktail (1:1000, ab110413), mouse ATP5A (1:1000, ab14748), rabbit anti-POLγA (1:250, ab12899), rabbit anti-TFAM (1:1000, ab131607), mouse anti-VDAC (1:1000, ab14734), all from Abcam, and rabbit anti-SSBP1 (1:500, HPA002866) from Sigma. Rabbit polyclonal antisera were generated by Agrisera and recognize the mouse TWINKLE, TFB2M, LRPPRC, and POLRMT proteins [48,56,57]. All TWINKLE, TFB2M, and POLRMT rabbit polyclonal antisera were affinity purified using the corresponding recombinant protein. The following secondary antibodies were applied at a 1:10,000 dilution: donkey anti-rabbit IgG (NA9340V) and sheep anti-mouse (NxA931) both from GE Healthcare. Detection of HRP-conjugated secondary antibodies was achieved by enhanced chemiluminescence (Immun-Star HRP Luminol/Enhancer from Bio-Rad).

### Glycerol gradients

Mitochondrial isolation from cells and glycerol density gradients were performed as described [58]. In brief, crude mitochondria were isolated from cells by differential centrifugation, treated with DNase and RNase and purified in sucrose gradients. A 15–40% glycerol gradient containing a 20% glycerol / 30% iodixanol pad at the bottom was cast using a gradient master (model 107, Biocomp) with the following settings: 2 min and 31 sec run time, 81.65 angle, and speed set to 14. Mitochondria (250–600 μg) were lyzed in 2% Triton X-100 for 15 min on ice and afterwards the total lysate was carefully layered on top of the gradient. Samples were centrifuged at 151,000 x g at 4°C for 4 hours. The gradient was manually fractionated in 750 μl increments from top to bottom. Fractions were then divided in half, whereby one portion was used to precipitate proteins overnight with 12% trichloroacetic acid and 0.02% sodium deoxycholate, followed by centrifugation at 20,000 x g for 20 min at 4°C and acetone washes, while the other half of fraction 1 was used to isolated mtDNA by proteinase K treatment followed by phenol/chloroform extraction. Thereafter, protein samples were processed for western blot analysis and mtDNA samples for Southern blot analysis.

### Determination of cellular dNTP pools

Cells were grown on 100 mm dishes in DMEM GlutaMax containing 25 mM glucose and supplemented with 10% FBS, 1% penicillin and streptomycin, 1% non-essential amino acids, and 50 μg/ml uridine. Positive control cells were treated with hydroxyurea (Sigma, H8627) at 2 mM for 30 hours to mildly deplete purines. Cells were quickly washed with ice-cold PBS, detached by trypsinization with 0.05% trypsin and the total number of cells was determined using a Vi-cell XR cell analyzer (Beckman Coulter). Approximately 2 million cells were centrifuged at 800x*g* for 5 min, washed with ice-cold PBS, and resuspended in 1 ml of ice-cold 60% methanol. Samples were then vortexed rigorously, frozen for 30 min at -20°C, and sonicated for 15 min on ice. After sonication, samples were centrifuged at 1000x*g* for 5 min at 4°C and supernatants collected. Extraction solution was evaporated using an Eppendorf Concentrator plus at room temperature.

Dried pellets were dissolved in 100 μl Milli-Q water, vortexed and sonicated for 2 min. After sonication samples were vortexed again and filtrated through a 0.2 μm modified nylon centrifugal filter (VWR) with an Eppendorf centrifuge 5424R set to 8°C and 12000 rpm. The external standard calibration curve was prepared in concentrations from 5 to 600 ng/ml of the dNTPs. All solutions were daily fresh prepared from stock solutions of 100 μg/ml and dissolved in Milli-Q water. dNTP analysis was conducted using a Dionex ICS-5000 (Thermo Fisher) Anion exchange chromatography using a Dionex Ionpac AS11-HC column (2 mm x 250 mm, 4 μm particle size, Thermo Fisher) at 30°C. A guard column, Dionex Ionpac AG11-HC b (2 mm x 50 mm, 4 μm particle size, Thermo Fisher), was placed before the separation column. The eluent (KOH) was generated in-situ by a KOH cartridge and deionized water. At a flow rate of 0.380 mL/min a gradient was used for the separation: 0–3 min 10 mM KOH, 3–12 min 10–50 mM, 12–19 min 50–100 mM, 19–21 min 100 mM 21–25 min 10 mM. A Dionex suppressor AERS 500, 2 mm was used for the exchange of the KOH and operated with 95 mA at 15°C. The suppressor pump flow was set at 0.6 mL/min. 10 μL of sample in a full loop mode (overfill factor 3) was injected. Autosampler was set to 6°C. The Dionex ICS-5000 was connected to a XevoTM TQ (Waters) and operated in negative ESI MRM (multi reaction monitoring) mode. The source temperature was set to 150°C, desolvation temperature was 350°C and desolvation gas was set to 50 L/h, cone gas to 50 L/h. The following MRM transitions were used for quantification: dGTP, transition 505.98 → 408.00, cone 30, collision 20; dATP, transition 490.02 → 158.89, cone 30, collision 26; dTTP, transition 480.83 → 158.88, cone 28, collision 46; dCTP, transition 465.98 → 158.81, cone 28, collision 30. The software MassLynx and TargetLynx (Waters) were used for data management and data evaluation & quantification. The calibration curve presented a correlation coefficient: r2 > 0.990 for all the compounds (response type: area; curve type linear). Quality control standards were tested during the sample analysis. The deviation along the run was between 0.5% and 40% respectively. Blanks after the standards, quality control and samples did not present significant peaks.

### Northern blotting

RNA was isolated from heart tissue either by using the ToTALLY RNA isolation kit (Ambion) or by using TRIzol Reagent (Invitrogen), and subjected to DNase treatment (TURBO DNA-free, Ambion). 1–2 μg of total RNA was denatured in RNA Sample Loading buffer (Sigma), electrophoresed in 1 or 1.8% formaldehyde-MOPS agarose gels prior transfer onto Hybond-N+ membranes (GE Healthcare). After UV crosslinking the membranes were successively hybridized with various probes at 65°C in RapidHyb buffer (Amersham) and then washed in 2x SSC/0.1% SDS and 0.2x SSC/0.1% SDS before exposure. Mitochondrial probes used for visualization of mRNA and rRNA levels were restriction fragments labeled with α-^32^P-dCTP and a random priming kit (Agilent). Different tRNAs and 7S rRNA were detected using specific oligonucleotides labeled with γ-^32^P-dATP. Radioactive signals were detected by autoradiography.

### mRNA expression analysis

Total RNA from frozen heart tissue was isolated using RNeasy Mini Kit (Qiagen) following the manufacturer’s instructions, DNase treated using TURBO DNA-free Kit (Thermo Fisher Scientific), and RNA subjected to reverse transcription PCR (RT-PCR) for cDNA synthesis using High Capacity cDNA reverse transcription kit (Applied Biosystems). Real-time quantitative reverse transcription PCR (qRT-PCR) was performed using the following Taqman probes from Thermo Fisher Scientific: *Mfn1* (Mm01289372_m1), *Mfn2* (Mm00500120_m1), and β-2-microglobulin (*B2M*, Mm00437762_m1). The quantity of transcripts was normalized to *B2M* as a reference gene transcript.

### mtDNA and 7S DNA quantification

#### mtDNA analysis by Southern blotting

Southern blotting was performed as previously described [48]. In brief, total DNA from heart tissue was extracted using phenol/chloroform. Total DNA, 5 μg, was digested with *Sac*I (New England Biolabs), the fragments were separated in a 0.8% agarose gel, and transferred to nitrocellulose membrane (Hybond-N+, GE Healthcare). Membranes were hybridized with α-^32^P-dCTP-labeled probes for mtDNA (pAM1) and nuclear DNA (18S).

#### D-loop Southern blotting

Mitochondrial DNA was isolated from crude heart mitochondria by phenol/chloroform extraction followed by RNase A treatment for 1 hour at 37°C. The mtDNA, 3 μg, was linearized overnight by SacI digestion at 37°C. DNA was precipitated, resuspended in 10 mM Tris pH 8.0, separated in a 0.9% agarose gel, transferred to a nitrocellulose membrane, and hybridized with α-^32^P-dATP-labeled probe recognizing mtDNA and 7S DNA.

#### mtDNA and 7S DNA analysis by quantitative PCR

Total DNA from heart tissue and immortalized MEFs was extracted using the DNeasy Tissue and Blood Kit (QIAGEN) based on the manufacturer’s instructions. Total DNA, 5 ng, was used to quantify mtDNA and nuclear DNA by qPCR using the following Taqman probes from Thermo Fisher Scientific: 16S (Mm04260181_s1), ATP6 (Mm03649417-g1), β-actin (Mm01205647_g1), and 18S (Hs99999901_s1). To quantify the mitochondrial 7S DNA, a custom Taqman probe recognizing mouse 7S DNA was constructed and validated using models known to have changes in 7S DNA levels. The mouse 7S DNA probe sequences are the following: forward primer, CTAATGTTATAAGGACATATCTGTGTTATCTGACATAC; reverse primer, GGTTTCACGGAGGATGGTAGATTAA; FAM reporter primer binding to 7S DNA and the displaced mtDNA strand, ACCATACAGTCATAAACTCTT. Since this probe can quantify both mtDNA (displaced strand) and 7S DNA, the abundance of 7S DNA was determined by normalizing the value obtained from 7S DNA probe to the value from an ATP6 probe which does not recognize 7S DNA.

### Mutation load analysis

Total DNA was extracted from heart and the somatic mtDNA mutation load was determined by post-PCR, cloning, and sequencing as described previously [59]. We used primers that amplify a region of the mtDNA spanning the 3’ end of *mtND2* through approximately a third of *mtCO1* (nucleotide pair 4950–5923 of the mouse reference mtDNA sequence GenBank NC_005089). The resulting clones were filtered to remove sequences derived from the known nuclear mitochondrial sequences (NUMTs).

### Sequencing to identify mitochondrial DNA deletions

DNA from isolated heart mitochondria was used to generate libraries for sequencing to detect mtDNA deletions. Total genomic DNA from the Deletor mouse was provided by Dr. Anu Suomalainen-Wartiovaara and used as a positive control for the detection of mtDNA rearrangements [32]. A standard Illumina TrueSeq paired-end library was prepared with ~500 base pair fragment inserts. Paired-end 100 base pair sequencing was conducted using an Illumina HiSeq 2500. The reads were mapped to the genomic sequence without the mitochondria using bowtie (VN: 2.1.0) to remove nuclear-genomic sequences [60]. The unmapped reads were then mapped to the mitochondrial sequence (GenBank JF286601.1) using bwa (n = 0.04, VN: 0.6.2-r126) [61] with unmapped reads undergoing an additional mapping round after trimming fastx_trimmer, VN: 0.0.13.2) to ensure higher mapping results. Using samtools [62] (VN: 1.0: samtools -f1 –F14) reads, where the two paired sequences were observed to be greater than 600 base pair apart were identified as those containing deletion breakpoints. These reads were extracted for additional analysis. The data presented in this publication have been deposited in NCBI's Gene Expression Omnibus [63] and are accessible through GEO Series accession number GSE124420 (https://www.ncbi.nlm.nih.gov/geo/query/acc.cgi?acc=GSE124420).

### mtDNA topology analysis

Total DNA was extracted from 20 mg of mouse heart tissue. Briefly, the minced tissue was lyzed in 600 μl of lysis buffer (100 mM Tris-HCl pH 7.5, 100 mM EDTA, 100 mM NaCl, 0.5% SDS, 0.5 mg/ml Proteinase K) at 55°C for 3 hours followed by 2 hours incubation on ice with premixed LiCl and K-acetate (final concentration 270 mM K-acetate, 820 mM LiCl) to precipitate contaminants. To remove the precipitate, the sample was centrifuged for 10 min at 10,000 rpm at room temperature and thereafter the DNA was precipitated with isopropanol overnight. The pelleted DNA was washed with 75% ethanol and resuspended to 10 mM Tris-HCl, 1 mM EDTA pH 8.0 followed by quantification with Qubit. To analyze the major topological isomers of the mtDNA, 200 ng of total DNA was resolved in a 0.4% agarose gel (15 x 15 cm) without ethidium bromide at 35 V for 20 hrs, followed by transfer onto Hybond-N+ membrane (GE Healthcare). The mtDNA was detected using [α-^32^P]-dCTP labeled probe (pAM1). To identify different topological isomers of the mtDNA, 200 ng of control total DNA was incubated at 37°C for 30 min with only buffer, SacI (New England Biolabs; 20 U), Nt.BbvCI (New England Biolabs; 10 U), Topo I (New England Biolabs; 5 U), Topo II (USB Affymetrix; 20 U), DNA gyrase (New England Biolabs; 5 U). The method was modified from [64].

### *de novo* mitochondrial transcription assay

Isolated heart mitochondria were purified by differential centrifugation in 320 mM sucrose, 10 mM Tris-HCl and 2 mM EDTA. From the same mitochondrial preparation: i) 300 μg of mitochondria were labeled with 50 μCi of [α-^32^P]-UTP (Perkin-Elmer) and processed as previously described to assess *de novo* mitochondrial transcription [57]; ii) as a loading control, 30 μg of mitochondria were subjected to SDS-PAGE and immunoblotted using mouse anti-VDAC antibody (Calbiochem); and iii) 300 μg of mitochondria were used to measure the ratio between 7S DNA and mitochondrial DNA. In the latter case, the mitochondrial pellet was resuspended in 50 mM Tris-HCl pH 7.5, 75 mM NaCl, 6.25 mM EDTA, 1% SDS and 1,2 mg/ml of Proteinase K and incubated for 1 hr at 37°C. After boiling for 5 min at 95°C, samples were electrophoresed in 0.8% agarose gels and transferred onto nylon membranes by Southern blotting. Both, mitochondrial DNA and 7S DNA were detected using [α-^32^P]-dCTP-labeled DNA probes specific for 7S DNA.

### *de novo* mitochondrial replication assay

Isolated heart mitochondria were purified by differential centrifugation and 15 μg of mitochondria were used to assess loading. For *de novo* mtDNA replicaton, 300 μg of mitochondria were washed with 500 μl ice-cold incubation buffer (25 mM sucrose, 75 mM sorbitol, 10 mM K_2_HPO_4_, 100 mM KCl, 0.05 mM EDTA, 5 mM MgCl_2_, 10 mM Tris-HCl pH 7.4, 10 mM glutamate, 2.5 mM malate, 1 mg/ml BSA, and fresh 1 mM ADP, final pH 7.2), centrifuged at 9,000 rpm for 4 min at 4°C, resuspended in incubation buffer containing 50 μM of dCTP, dGTP, dTTP and 20 μCi of radioactive α-^32^P-dATP, and incubated for 2 hours at 37°C. Thereafter, mitochondria were centrifuged at 9,000 rpm for 4 min at 4°C and washed 3x with 500 μl ice-cold washing buffer (10% glycerol, 0.15 mM MgCl_2_, 10 mM Tris-HCl pH 6.8). Mitochondria were treated with 1,2 mg/ml proteinase K and DNA extracted with phenol/chloroform. The mtDNA was resuspended in 30 μl TE buffer and one half boiled at 95°C for 5 min. Next, DNA was separated by electrophoresis on a 0.8% agarose gel for 15 hours at 20 V and transferred onto nylon membranes (Hybond-N+, GE Healthcare), followed by membrane cross-linking. Both Phosphor Imager (Fuji Film FLA-7000) and autoradiography films (GE Healthcare) were used to detect the radioactive signal. After the radioactive signal had decayed, steady-state mitochondrial DNA and 7S DNA levels were assessed by using an α-^32^P-dATP-labeled DNA probe for 7S DNA.

#### Analysis of abortive mtDNA products

*de novo* mtDNA replication images were quantified using ImageJ for densitometry analysis. The cumulative abortive mtDNA products were defined as the signal directly above and below the 7S DNA band.

### Growth curve assay

The growth rate of log phase cells was assessed by equilibrating cells to galactose media (DMEM supplemented with 15 mM galactose, 1mM sodium pyruvate (Thermo Fisher Scientific, 11360–039), 10% FBS, 1% Penicillin/Streptomycin, 1% non-essential amino acids, and 50 μg/ml uridine) for 3 days, followed by plating 27,000 cells in a six-well plate containing 3 ml of galactose media. Cells were collected by trypsinization with 1 ml of 0.05% Trypsin-EDTA (Thermo Fisher Scientific, 25300–054) every 24 hours and viable cells counted using a Vi-Cell XR analyzer (Beckman Coulter).

### Cellular respiration

MEFs in log phase were grown in DMEM GlutaMax containing 25 mM glucose and supplemented with 1% dialyzed fetal bovine serum, 1% penicillin/streptomycin, 1% non-essential amino acids, and 50 μg/ml uridine for 5 to 6 days. MEFs were passaged once during this timeframe. Cells were then collected by trypsinization and counted. The flux of mitochondrial oxygen consumption was determined using 1 million viable cells as described for isolated heart mitochondria, see *Mitochondrial respiration*, expect for the following modifications. Cells were permeabilized with 0.02 mg/ml digitonin and the oxygen consumption rate in state 3 was assessed using 10 mM succinate and 5 mM glycerol-3-phosphate.

### Ethidium bromide treatment of cells

MEFs were cultivated in 18 ml of DMEM GlutaMax containing 25 mM glucose and supplemented with 10% FBS, 1% penicillin and streptomycin, 1% non-essential amino acids, and fresh 100 ng/ml ethidium bromide. After 6 days of cultivation in the presence of ethidium bromide, cells were switched to medium containing no ethidium bromide for 6 days. Cells were passaged every 2–3 days during both conditions. Total DNA was extracted using the DNeasy Tissue and Blood Kit (QIAGEN) and mtDNA levels determined, see *mtDNA quantification by qPCR*.

### Immunocytochemistry

#### Cell lines

Cells plated onto glass coverslip were fixed in 4% paraformaldehyde for 10 min at room temperature, washed twice with PBS, permeabilized with 0.1% Triton-X in PBS for 5 min, blocked in 3% BSA for 30 min, and incubated overnight at 4°C with the following primary antibodies prepared in 3% BSA: mouse IgM anti-DNA (Progen, 61014, 1:250), rabbit anti-TOM20 (Santa Cruz, sc-11415, 1:1000), goat anti-HSP60 (Santa Cruz, 1052, 1:500), rabbit anti-SSBP1 (Sigma, HPA002866 1:500), rat anti-BrdU (Abcam, 6326,1:250), and rabbit anti-TFAM (Agrisera, custom-made, 1:3000). The following secondary antibodies at 1:500 in 3% BSA were incubated for 2 hours at room temperature: donkey anti-mouse IgM Alexa 488, donkey anti-goat Alexa 633, donkey anti-rat DyLight 488, or donkey anti-rabbit Cy3. Coverslips were extensively washed with PBS, counterstained with DAPI in PBS (1:1000, Invitrogen) before mounting with Aqua/Poly-mount (Polyscience Inc.).

For super-resolution imaging, cells were grown on coverslips overnight, fixed with methanol at -20°C for 5 min, blocked with 5% (w/v) BSA in PBS for 5 min, incubated with 20 μg/ml RNAse A (Sigma Aldrich) in 0.5% Tween 20 (Sigma Aldrich) in PBS for 2 hours at 37 °C. Finally, the cells were incubated with Quant-iT PicoGreen dsDNA Reagent (Thermo Fisher Scientific) in PBS for 30 min. After several washing steps, the samples were mounted in Mowiol. Samples were measured within three hours after the sample preparation.

#### Heart tissue

Freshly isolated hearts were fixed by immersion in 4% PFA, cryopreserved in 30% sucrose and frozen in optimal cutting temperature compound (OCT). 10 μm-thick cryosections were cut and air dried prior to fluorescent immunohistochemistry. Sections were then permeabilized in 0.5% Triton X-100/PBS and binding of antibodies to unspecific sites was prevented by incubation in blocking buffer (3% BSA/PBS). Primary antibody incubation was performed overnight at 4°C, by applying the following antibodies in blocking buffer: mouse IgM anti-DNA (Progen, 1:100), chicken anti-GFP (Aves, 1:300). After washing, the following secondary antibodies were applied: goat anti-mouse IgM Alexa 594, and donkey anti-chicken Alexa 488. Nuclei were counterstained with DAPI.

### Fluorescence *in situ* hybridization

For experiments requiring dual visualization of mRNA and proteins, FISH was performed prior to immunocytochemistry, by using the QuantiGene ViewRNA ISH Cell Assay (Affymetrix) following the manufacturer’s instructions. To visualize mitochondrial RNA, cells were hybridized 3 hours at 40°C using the *Cox1*mRNA probe (1:500, cat# VB4-17017, Affymetrix). To confirm probe specificity towards RNA, treatments with RNAse T1 and DNAse1 (both at 250U/mL, 37°C) were performed prior to hybridization.

### Bromouridine labeling

Cells grown on coverslips for 24 hours in DMEM medium, without uridine supplementation were incubated with 5 mM BrU for 1 hour at 37°C and 5% CO_2_. A short chase using DMEM medium containing 50 μg/ml uridine was performed followed by a PBS wash prior to fixation. BrU was prepared fresh for each experiment. Standard immunocytochemistry procedures were carried out to visualize the mitochondrial network, mtDNA, and newly synthesized BrU-labeled RNA.

### Imaging of MEFs

#### Confocal microscopy

Fluorescence images were acquired using a laser-scanning Leica TCS SP8-X inverted confocal microscope, containing a white light laser, a 405-diode UV laser, and a 100x objective (HCX PL APO 100x oil, 1.46 N.A). Stack images were acquired in sequential mode using a scanning speed of 200 Hz, an image size of 1,024 x 1,024 pixels, and z-step of 0.2 μm.

#### STED microscopy

STED images of MEFs were acquired with a two-color Abberior STED 775 QUAD scanning nanoscope (Abberior Instruments, Goettingen Germany) using a 485 nm excitation laser and a pulsed 595 nm STED laser (PicoGreen labeling) or a 640 nm excitation laser together with a 775 nm STED laser (Antibody labeling) and a 100x oil immersion objective lens (NA 1.4). Image analysis was performed manually using Imspector Software, by measuring the diameter of nucleoids at full width at half maximum (Abberior Instruments, Goettingen Germany).

### Imaging of heart tissue

Imaging of heart sections was performed using a Leica TCS SP8 gated STED (gSTED) microscope, equipped with a white light laser and a 93x objective lens (HC PL APO CS2 93x GLYC, NA 1.30). For confocal images of mitochondria and DNA, Z-stacks in accordance with the Nyquist sampling criteria were taken by exciting the fluorophores at 488 nm and 594 nm, respectively, and Hybrid detectors collected fluorescent signals. Stimulated emission depletion of DNA channel was performed with a 775 nm depletion laser. 2D confocal and gSTED images were acquired sequentially with the optical zoom set to obtain a voxel size of 17 x 17 nm. Excitation was provided at 594 nm and Hybrid detectors collected signal. Gating between 0.3–6 ns was applied. Images were deconvolved with the Huygens software. Performance of the microscope and optimal depletion laser power were tested as previously described [65].

### Image analysis

#### Image analysis of *CoxI* mRNA and BrU-labeled RNA in MEFs

Analysis of *Cox1*mRNA and BrU-labeled RNA in MEFs was performed in ImageJ. For quantification of *Cox1*mRNA and the BrU-labeled RNA per cell, a ROI corresponding to the cell boundaries was drawn, and the DAPI channel was used to exclude the nuclear region prior to analysis. Integrated density of the signal coming from the *Cox1*mRNA channel or the BrU channel was then quantified and normalized over the cytoplasmic area. For quantification of nucleoids positive for *Cox1* mRNA or BrU-labeled RNA, dual color pictures of DNA, *Cox1*mRNA, and BrU channels were generated in ImageJ. Nucleoids were then classified based on the presence or absence of *Cox1*mRNA foci or BrU foci in their proximity and quantified using the Cell Counter plug-in from ImageJ.

#### Image analysis of nucleoid and SSBP1 abundance in MEFs

The amount mtDNA foci per cell was determined using stacked confocal images acquired and analyzed using ImageJ. Prior to analysis, the DAPI channel was used to manually trace nuclear DNA with the polygon section tool, a region of interest (ROI) selected, and this same area omitted from the anti-DNA channel to quantify only mtDNA foci for each cell. The find maxima tool was used to count mtDNA and SSBP1 foci with noise tolerance manually determined between 50–60. To determine the relative abundance of mtDNA foci relative to TOM20 surface area, binary images were generated from stacked confocal images. A ROI encompassing the mitochondrial network, as visualized by immunostaining for TOM20, was selected for each cell and the analyze particles tool was used to determine the total surface area. The same ROI was then applied to the anti-DNA image with an omitted nuclear DNA area to determine the total number of mtDNA particles.

#### Line scan analysis of SSBP1-mtDNA in MEFs

A line scan analysis of SSBP1 was performed on stacked confocal images acquired and analyzed using ImageJ. Using merged images of SSBP1, mtDNA, and HSP60, a line segment was drawn larger than the diameter of the SSBP1 foci, the RGB profiler plugin applied, and spectra plots analyzed. SSBP1 spectra plots were categorized into three groups; no overlap with mtDNA (separated), partial overlap with mtDNA, and full overlap with mtDNA.

#### Image processing

Image panels were assembled with Photoshop (Adobe); no digital manipulation was applied except for unbiased adjustment of brightness and contrast.

### Quantification and statistical analysis

Data are presented as mean ± SEM unless otherwise indicated in figure legends. Sample number (n) indicates the number of independent biological samples (individual mice, number of cells, or wells of cells) in each experiment. Sample numbers and experimental repeats are indicated in the figures. Data were analyzed in Graphpad Prism using the unpaired Student’s t-test, one-way ANOVA using Turkey’s multiple comparison test, two-way ANOVA using Bonferroni multiple comparison test between group comparison, as appropriate. A p-value ≤ 0.05 was considered statistically significant.

## Supporting information

S1 Fig Genetic and functional characterization, related to Fig 1. (A) Genotype distribution of progeny born from seven intercrosses (n = 52) between *Mfn1*^*loxP/ loxP*,^ *Mfn2*^*loxP/ loxP*^ females and *Mfn1*^*loxP/ loxP*^, *Mfn2*^*loxP/+*^, Ckmm-*Cre*^*+/-*^ males. (B) RT-PCR quantification of transcripts in heart from control (n = 4) and *dMfn* KO (n = 5) animals at 4 weeks of age. Normalization to beta-2-microglobulin. (C) Assessment of cellular respiration from control (n = 5), *dMfn* KO (n = 6), *Opa1* KO (n = 6) MEFs. Cells were grown in glucose medium with 1% dialyzed FBS (dFBS) for 5–6 days, permeabilized and mitochondrial respiration was assessed under phosphorylating (ST3), non-phosphorylating (ST4), and uncoupled (UC) conditions using complex II substrates. (D) Growth curves of control, *dMfn* KO, and *Opa1* KO MEFs grown in DMEM medium with galactose. For all genotypes three independent experiments, each with three technical replicates, were performed. For all, error bars indicate ± SEM. (B) Student T-test; ***, P < 0.001. For (C) and (D), two-way ANOVA using Bonferroni multiple comparison test was used; ***, P < 0.001.
(TIF)

S2 Fig Characterization of nucleoid distribution, related to Fig 3. (A) Quantification of nucleoids per cell in control and *dMfn* KO MEFs. Total nucleoids were counted from stacked confocal images decorated with anti-DNA antibodies. In total, 3 independent experiments were performed for each genotype and 9–11 cells measured per experiment. (B) Quantification of nucleoids (mtDNA foci) per mitochondrial surface area (TOM20) in control and *dMfn* KO MEFs. Total mtDNA foci and the mitochondrial surface area were determined from stacked confocal images. In total, 3 independent experiments were performed for each genotype and 9–11 cells measured per experiment. For A and B, Error bars indicate ± SEM. Student T-test; ***, P < 0.001. (C) Representative images of control and *dMfn* KO MEFs labeled with anti-DNA antibodies and imaged by confocal and STED microscopy. Scale bar is 1 μm. (D) Quantification of the average nucleoid diameters in confocal and STED acquired images after labeling with anti-DNA antibodies in control, *Mfn1* KO, *Mfn2* KO, *dMfn* KO, *Opa1* KO MEFs. The nucleoid diameters were measured at full width at half maximum on 100 nucleoids from each genotype. (E) Quantification of the ratio between the nucleoid diameters observed by confocal and STED images acquired after anti-DNA labeling in control, *Mfn1* KO, *Mfn2* KO, *dMfn* KO, and *Opa1* KO MEFs, n = 12 for all genotypes. Errors bars indicate the standard error of the mean. For (D and E), one-way ANOVA using Turkey’s multiple comparison test; *, P < 0.05; ***, P < 0.001.
(TIF)

S3 Fig Abundance of mitochondrial transcripts, 7S RNA and 7S DNA, related to Fig 5. (A) Northern blot analysis of mitochondrial transcripts from the heavy and light strand promoter (HSP and LSP) of control and *dMfn* heart KO animals at 5 weeks of age (n = 4 for each genotype). *Mterf4* and *Polrmt* heart knockouts were included as controls for increase and decrease of mtDNA transcription. (B) Quantification of 7S RNA abundance relative to nuclear DNA (18S) as determined by northern blot analysis, related to (A), n = 4 for each genotype. (C) Southern blot quantification of relative 7S DNA levels in heart mitochondria from controls and *dMfn* KO mice, n = 4 for both genotypes. (D) Quantitative PCR analysis of 7S DNA levels relative to mtDNA levels (ATP6) in heart tissue from control (n = 3) and *dMfn* KO (n = 3) at 3 weeks of age; control (n = 4) and *Mgme1* KO (n = 4) at 11 weeks of age; and control (n = 4) and *Polrmt* KO (n = 4) animals at 4 weeks of age. Error bars indicate ± SEM. For all Student T-test; *, P < 0.05; ***, P < 0.001; n.s, no significant difference.
(TIF)

S4 Fig Abundance of cellular dNTP pools in MEFs, related to Fig 6. (A) Quantification of cellular dNTPs by UPLC-MS from control (n = 10) and *dMfn* KO (n = 11), *Opa1* KO (n = 12) MEFs, control MEFs without hydroxyurea treatment (-HU, n = 3), and control MEFs treated with for 30 hours with hydroxyurea (+HU, n = 3) MEFs. A two-way ANOVA using Bonferroni multiple comparison test was performed and no statistical difference was observed between genotypes and deoxynucleotides.
(TIF)
