# Supplementary figures and images for: Mitochondrial fusion is required for regulation of mitochondrial DNA replication
Source: PLoS Genet. 2019 Jun 6;15(6):e1008085. doi: 10.1371/journal.pgen.1008085 (PMC6553695; doi:10.1371/journal.pgen.1008085)

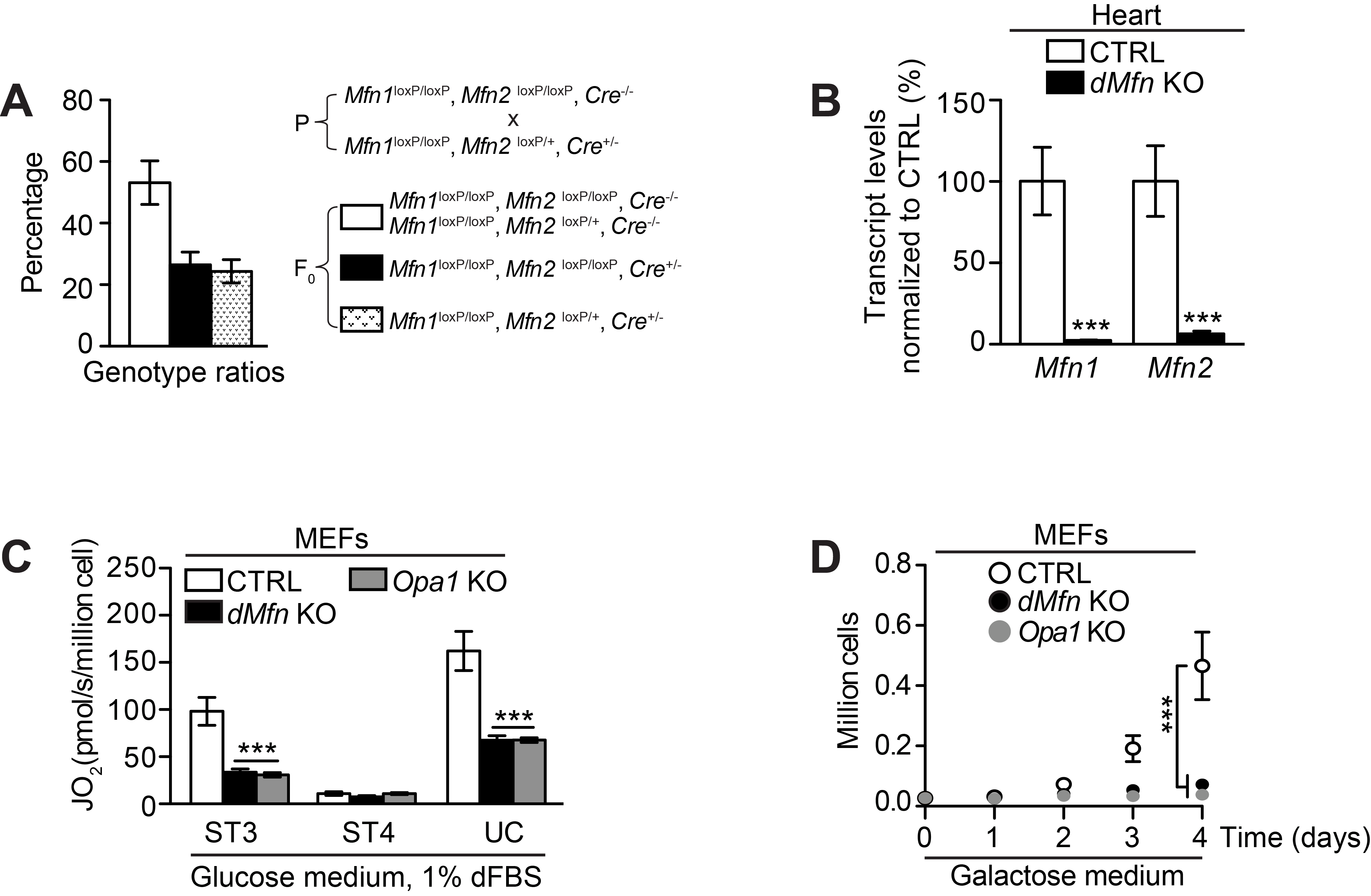

Supplement: S1 Fig — (A) Genotype distribution of progeny born from seven intercrosses (n = 52) between Mfn1loxP/ loxP, Mfn2loxP/ loxP females and Mfn1loxP/ loxP, Mfn2loxP/+, Ckmm-Cre+/- males. (B) RT-PCR quantification of transcripts in heart from control (n = 4) and dMfn KO (n = 5) animals at 4 weeks of age. Normalization to beta-2-microglobulin. (C) Assessment of cellular respiration from control (n = 5), dMfn KO (n = 6), Opa1 KO (n = 6) MEFs. Cells were grown in glucose medium with 1% dialyzed FBS (dFBS) for 5–6 days, permeabilized and mitochondrial respiration was assessed under phosphorylating (ST3), non-phosphorylating (ST4), and uncoupled (UC) conditions using complex II substrates. (D) Growth curves of control, dMfn KO, and Opa1 KO MEFs grown in DMEM medium with galactose. For all genotypes three independent experiments, each with three technical replicates, were performed. For all, error bars indicate ± SEM. (B) Student T-test; ***, P < 0.001. For (C) and (D), two-way ANOVA using Bonferroni multiple comparison test was used; ***, P < 0.001. (TIF) [file pgen.1008085.s001.tif]

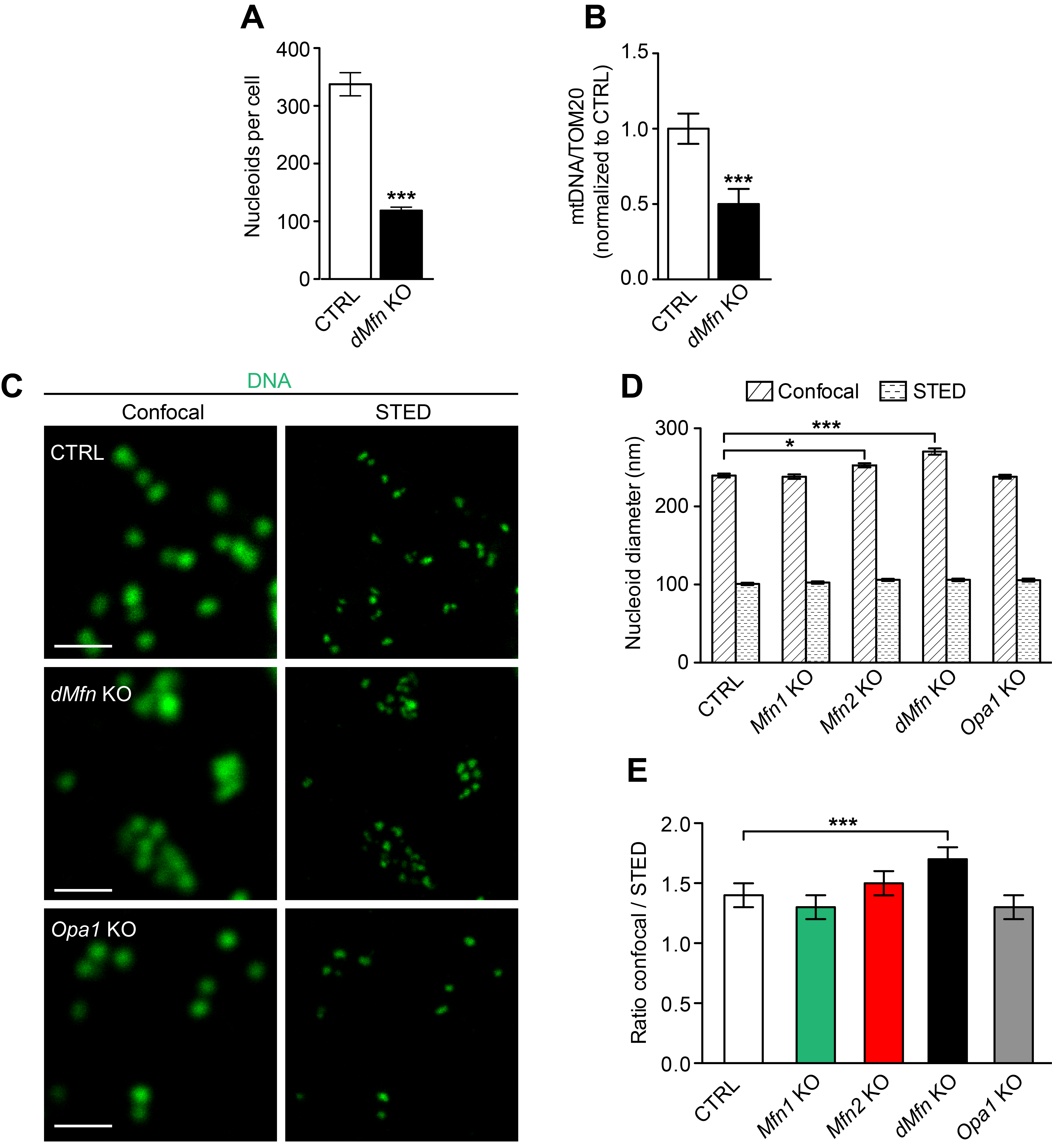

Supplement: S2 Fig — (A) Quantification of nucleoids per cell in control and dMfn KO MEFs. Total nucleoids were counted from stacked confocal images decorated with anti-DNA antibodies. In total, 3 independent experiments were performed for each genotype and 9–11 cells measured per experiment. (B) Quantification of nucleoids (mtDNA foci) per mitochondrial surface area (TOM20) in control and dMfn KO MEFs. Total mtDNA foci and the mitochondrial surface area were determined from stacked confocal images. In total, 3 independent experiments were performed for each genotype and 9–11 cells measured per experiment. For A and B, Error bars indicate ± SEM. Student T-test; ***, P < 0.001. (C) Representative images of control and dMfn KO MEFs labeled with anti-DNA antibodies and imaged by confocal and STED microscopy. Scale bar is 1 μm. (D) Quantification of the average nucleoid diameters in confocal and STED acquired images after labeling with anti-DNA antibodies in control, Mfn1 KO, Mfn2 KO, dMfn KO, Opa1 KO MEFs. The nucleoid diameters were measured at full width at half maximum on 100 nucleoids from each genotype. (E) Quantification of the ratio between the nucleoid diameters observed by confocal and STED images acquired after anti-DNA labeling in control, Mfn1 KO, Mfn2 KO, dMfn KO, and Opa1 KO MEFs, n = 12 for all genotypes. Errors bars indicate the standard error of the mean. For (D and E), one-way ANOVA using Turkey’s multiple comparison test; *, P < 0.05; ***, P < 0.001. (TIF) [file pgen.1008085.s002.tif]

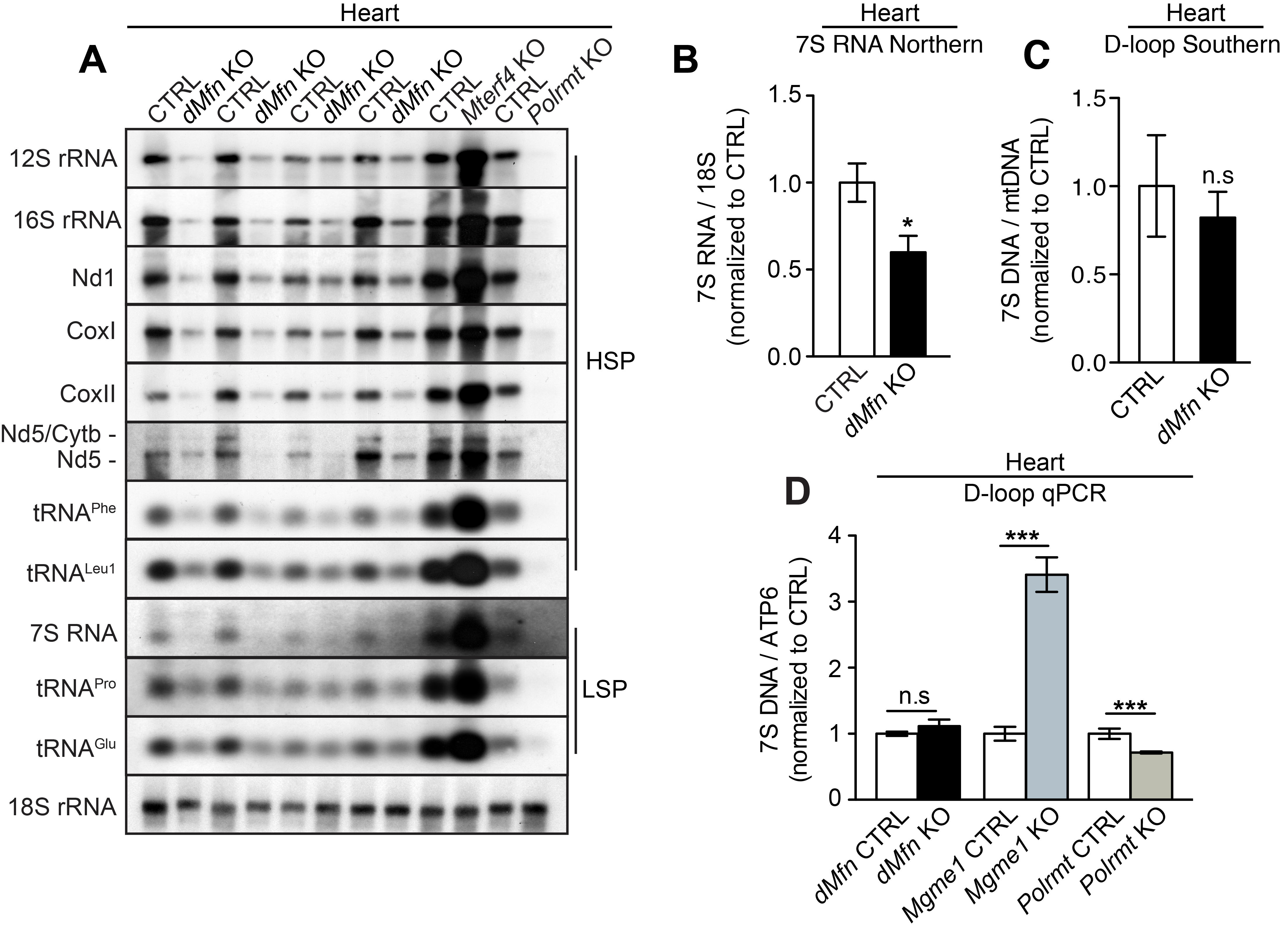

Supplement: S3 Fig — (A) Northern blot analysis of mitochondrial transcripts from the heavy and light strand promoter (HSP and LSP) of control and dMfn heart KO animals at 5 weeks of age (n = 4 for each genotype). Mterf4 and Polrmt heart knockouts were included as controls for increase and decrease of mtDNA transcription. (B) Quantification of 7S RNA abundance relative to nuclear DNA (18S) as determined by northern blot analysis, related to (A), n = 4 for each genotype. (C) Southern blot quantification of relative 7S DNA levels in heart mitochondria from controls and dMfn KO mice, n = 4 for both genotypes. (D) Quantitative PCR analysis of 7S DNA levels relative to mtDNA levels (ATP6) in heart tissue from control (n = 3) and dMfn KO (n = 3) at 3 weeks of age; control (n = 4) and Mgme1 KO (n = 4) at 11 weeks of age; and control (n = 4) and Polrmt KO (n = 4) animals at 4 weeks of age. Error bars indicate ± SEM. For all Student T-test; *, P < 0.05; ***, P < 0.001; n.s, no significant difference. (TIF) [file pgen.1008085.s003.tif]

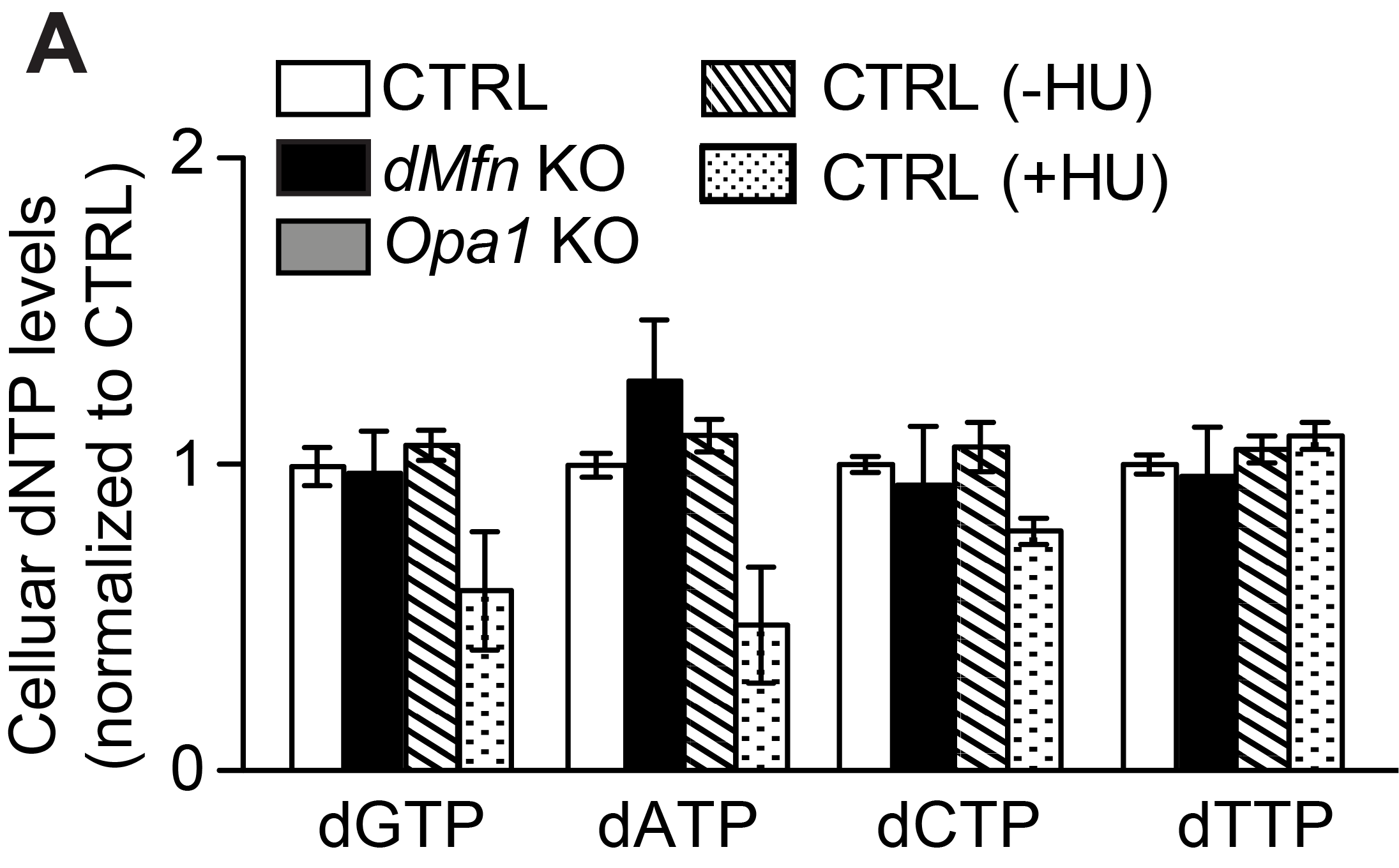

Supplement: S4 Fig — (A) Quantification of cellular dNTPs by UPLC-MS from control (n = 10) and dMfn KO (n = 11), Opa1 KO (n = 12) MEFs, control MEFs without hydroxyurea treatment (-HU, n = 3), and control MEFs treated with for 30 hours with hydroxyurea (+HU, n = 3) MEFs. A two-way ANOVA using Bonferroni multiple comparison test was performed and no statistical difference was observed between genotypes and deoxynucleotides. (TIF) [file pgen.1008085.s004.tif]
